# Actions on sustainable food production and consumption for the post-2020 global biodiversity framework

**DOI:** 10.1126/sciadv.abc8259

**Published:** 2021-03-19

**Authors:** Izabela Delabre, Lily O. Rodriguez, Joanna Miller Smallwood, Jörn P. W. Scharlemann, Joseph Alcamo, Alexander S. Antonarakis, Pedram Rowhani, Richard J. Hazell, Dag L. Aksnes, Patricia Balvanera, Carolyn J. Lundquist, Charlotte Gresham, Anthony E. Alexander, Nils C. Stenseth

**Affiliations:** 1 Sussex Sustainability Research Programme, University of Sussex, Brighton BN1 9SL, UK.; 2 University of Sussex Business School, University of Sussex, Brighton BN1 9SN, UK.; 3 International Union of Biological Sciences (IUBS), Bat 442, Université Paris-Sud 11, 91 405 Orsay Cedex, France.; 4 Centro de Conservación, Investigación y Manejo de Áreas Naturales–Cordillera Azul, Av. Benavides 1238 Of. 601, Lima 18, Peru.; 5 School of Life Sciences, University of Sussex, Brighton BN1 9QG, UK.; 6 School of Global Studies, University of Sussex, Brighton BN1 9SJ, UK.; 7 Department of Biological Sciences, University of Bergen, P.O. Box 7803, N-5020 Bergen, Norway.; 8 Instituto de Investigaciones en Ecosistemas y Sustentabilidad, Universidad Nacional Autónoma de México, Morelia, Michoacán 58350 Mexico.; 9 Unidad Académica de Estudios Territoriales. Universidad Nacional Autónoma de México, Oaxaca 68000 Mexico.; 10 National Institute of Water and Atmosphere Research (NIWA), Hamilton, New Zealand.; 11 Institute of Marine Science, University of Auckland, Auckland, New Zealand.; 12 Centre for Ecological and Evolutionary Synthesis (CEES), Department of Biosciences, University of Oslo, N-0316 Oslo, Norway.

## Abstract

Current food production and consumption trends are inconsistent with the Convention on Biological Diversity’s 2050 vision of living in harmony with nature. Here, we examine how, and under what conditions, the post-2020 biodiversity framework can support transformative change in food systems. Our analysis of actions proposed in four science-policy fora reveals that subsidy reform, valuation, food waste reduction, sustainability standards, life cycle assessments, sustainable diets, mainstreaming biodiversity, and strengthening governance can support more sustainable food production and consumption. By considering barriers and opportunities of implementing these actions in Peru and the United Kingdom, we derive potential targets and indicators for the post-2020 biodiversity framework. For targets to support transformation, genuine political commitment, accountability and compliance, and wider enabling conditions and actions by diverse agents are needed to shift food systems onto a sustainable path.

## Teaser

Actions to transform food systems are key to addressing biodiversity loss.

## INTRODUCTION

Food is an essential contribution from nature to people, ultimately underpinned by biodiversity (*1*, *2*). Yet, food systems are responsible for around 60% of global terrestrial biodiversity loss and the overexploitation of 33% of commercial fish populations (*3*, *4*). At the same time, one-third of all food goes to waste between the points of production and consumption, while around 11% of the world’s population is undernourished (*5*) and 39% are overweight or obese (*6*). The external costs of the food system are estimated at around US$12 trillion a year, rising to US$16 trillion by 2050 (*7*). The Intergovernmental Science-Policy Platform on Biodiversity and Ecosystem Services (IPBES) global assessment warned that biodiversity is declining faster than at any time in human history and that all contributions from nature to people are decreasing, except food provision at the expense of other contributions (*8*).

In its strategic plan 2010–2020, the United Nations Convention on Biological Diversity (CBD) recognized that to achieve its 2050 vision of “living in harmony with nature,” it was necessary to address food production and consumption, as major underlying causes of biodiversity loss. Shifting toward sustainable production and consumption is a cornerstone for mainstreaming, as stipulated within CBD Aichi Target 4 (*9*). It was stated that biodiversity conservation and sustainable use should be included in policies, strategies, and practices of key public and private actors that affect or rely on biodiversity, both locally and globally (*10*). Increased awareness of how the food system drives biodiversity change from a distance (through telecoupling, i.e., socioeconomic and environmental connections over distances) has made these trends evident and exposed their severity (*8*, *11*). The fifth Global Biodiversity Outlook acknowledged that CBD Aichi Target 4 related to sustainable production and consumption was not met nor were associated Targets 5, 6, and 7 referring to land-use change, fisheries, and sustainable use (*12*).

Although an opportunity has been missed in achieving objectives that connect biodiversity and food production and consumption in the 2010–2020 strategic period, a new opportunity is imminent. At the 15th Conference of the Parties (COP), CBD members will decide on the post-2020 global biodiversity framework. Recognizing the need to go beyond incremental change, the IPBES global assessment identified several “leverage points” for initiating transformations through multilevel governance interventions (“levers”), to influence values and behavior and address the direct and indirect drivers of biodiversity loss (*8*). Given the role of biodiversity in supporting the United Nations Sustainable Development Goals (SDGs), it is critical to consider how, and under what conditions, specific actions and actors in the food system can support sustainability transformation in social-ecological systems, paying attention to synergies, feedbacks, and unintended consequences (*13*, *14*). Important work has been undertaken to model scenarios for bending the curve on biodiversity loss, emphasizing the need for integrated strategies on food and climate (*15*). Research that engages explicitly with challenges in policy and governance is crucial to consider the complexities of implementation (*16*), and imagine how current mechanisms can better support transformative change.

Here, we focus on agriculture and fisheries because they are among the largest drivers of global environmental change in the Anthropocene, driven by rising global demand for food, fuel, and animal feed (*8*, *17*). In turn, these changes threaten food production and other contributions from nature to people (*18*, *19*). By 2050, the projected human population of 9.8 billion is predicted to require a 100 to 110% increase in global crop production compared with production in 2005, and to fulfill this demand, agricultural land or productivity (cropland and animal productivity) must increase (*20*–*23*). However, recent productivity increases have not kept up with increasing demand, suggesting that the continued expansion of agricultural land is inevitable (*24*, *25*), with diverse consequences on social-ecological systems and, in turn, on human health (*26*, *27*), and equity and justice (*28*, *29*). Furthermore, increases in cropland productivity likely come at the cost of increased pressure on natural ecosystems in the form of habitat loss, nutrient runoff, pesticide accumulation, and other impacts (*30*, *31*). Without shifts in consumption patterns, increased pressure on natural fish stocks is also expected, including in meeting demand for major feed ingredients (such as soy) used in fish and crustacean aquaculture. While considering actions for food production and consumption, it is important to note that data are challenging to disaggregate on impacts of agriculture for food production and for nonfood/nonfeed production.

Overfishing in capture fisheries (or “commercial fishing”) is one important issue for marine biodiversity, though marine ecosystems are also degraded by stressors such as coastal eutrophication (partly caused by agriculture), deoxygenation, ocean warming, and ocean acidification. To feed the world, it is necessary to think about how to optimally use terrestrial, aquatic and marine food systems. Although these food systems are frequently discussed separately, there is a need to consider how these food systems interact in relation to sustainability and how they link with the needs of producers and consumers (*32*).

The compounding effects of climate change on biodiversity loss (*33*, *34*), food systems (*35*), and human well-being (*36*, *37*), as well as impacts on water availability (*38*), present further complexities in the food-biodiversity domain. Climate change mitigation measures result in pressures on land use; likewise, the promotion of bioenergy crops, restoration, and afforestation affects biodiversity, food production, and water demand, as well as local livelihoods, food access and rights (*8*). Climate change affects biophysical processes and productivity, further aggravating existing vulnerabilities for food system actors (*39*). Although these interrelationships complicate biodiversity policy-making, they also provide opportunities for synergistic policies and measures. Ecosystem restoration of forests and other “high carbon” landscapes, for example, not only mitigates climate change but also regulates extreme hydrometeorological events, increases resilience, and enriches sources of micronutrients (*40*–*43*). Meanwhile, the restoration of oceanic and coastal ecosystems can trigger the recovery of fisheries, enhance food security, help secure livelihoods, and increase opportunities for ecotourism and carbon sequestration.

With the overarching goal to sustainably obtain sufficient food for people while conserving and restoring biodiversity, we address five questions that examine how, and under what conditions the post-2020 global biodiversity framework can support leverage points for transformative change, with a specific focus on food production and consumption: (i) What are the key actions proposed, related to production and consumption of food, in science-policy fora intended to inform the post-2020 biodiversity framework? (ii) To what extent are these key actions addressed in the Aichi Targets, the SDGs, the CBD Zero Draft, and Update? (iii) What barriers and opportunities exist in implementing the key actions, at the global level, and in relation to two country case studies: Peru and the United Kingdom? (iv) What potential targets and indicators could support a post-2020 biodiversity framework that effectively addresses sustainable food production and consumption? and (v) What are the enabling conditions needed to support the achievement of these targets?

## RESEARCH APPROACH

Our investigations (outlined steps; fig. S1) are based on systematic analyses of outputs from four science-policy fora intended to inform the post-2020 global biodiversity framework: the “IPBES visioning workshop,” New Zealand 2017; two fora organized by the International Union of Biological Sciences (IUBS): “4th Science Forum,” CBD/COP14 Egypt 2018 and the 100th General Assembly, Norway 2019; and the “9th Trondheim Conference on Biodiversity,” Norway 2019 (details in table S1). These fora were selected because they brought together diverse groups of policy-makers, private sector actors, and researchers from multiple disciplines. The groups examined a range of topics in environmental and social sciences, therefore representing diverse perspectives of food systems from beyond the biodiversity sector. We extracted direct text relating to actions (including research, innovation, policy, and management) specifically related to sustainable food production and consumption from output documents (full list in table S2). From relevant documents, we derive a “short list” of eight key actions that particularly mitigate the main direct causes of biodiversity loss—loss of habitat due to agricultural activities and unsustainable use of fisheries.

**Fig. 1:**
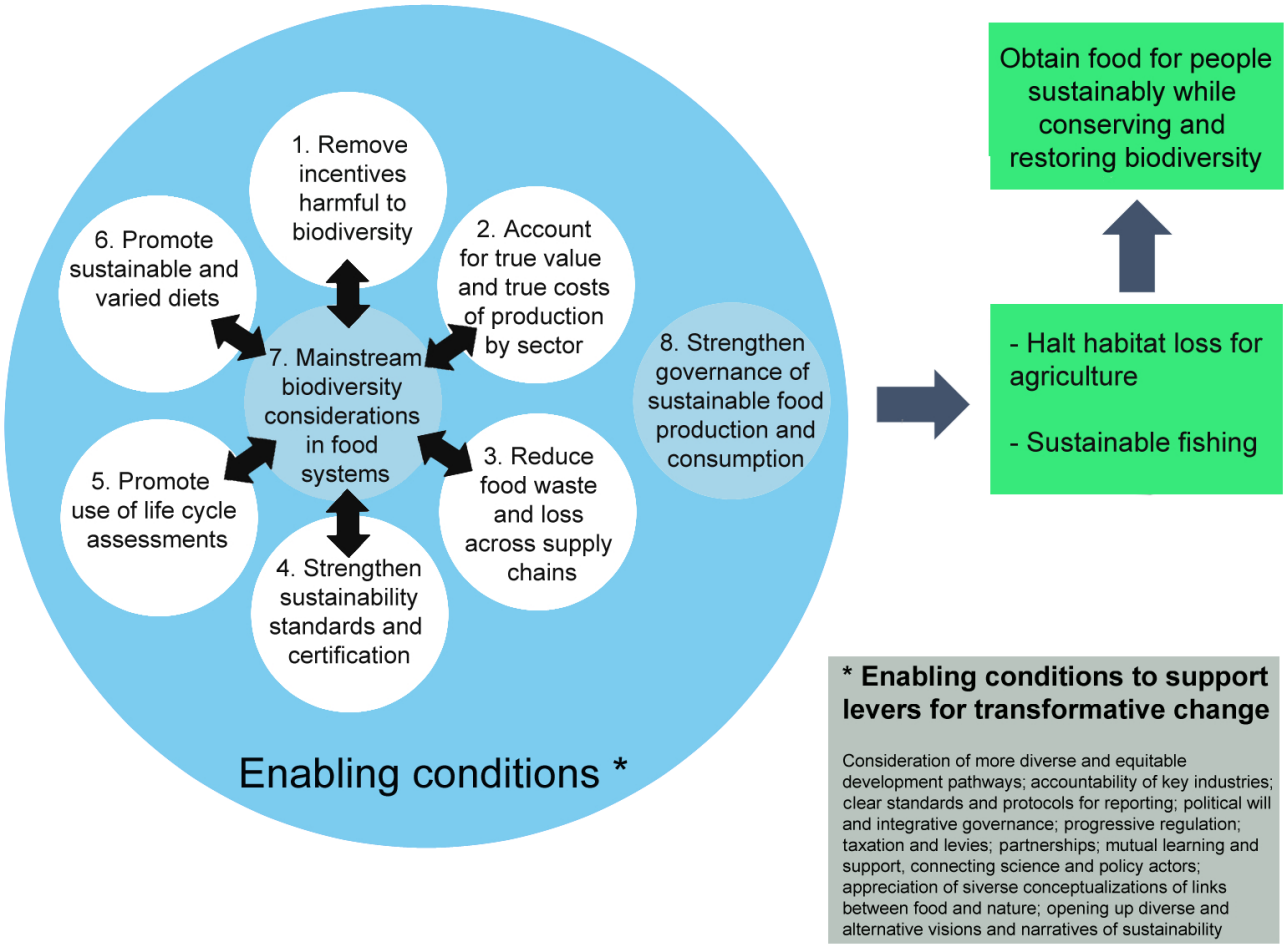
Proposed key actions and enabling conditions. Together, these can support a transformative post-2020 global biodiversity framework to achieve sustainable food production and consumption. Actions 7 and 8 are cross-cutting strategic actions, which affect or are affected by the implementation of all other actions. Action 7 includes integrating biodiversity into national and local planning, development processes, and poverty reduction and accounts, and Action 8 refers to strengthening governance of the sustainable production and consumption of food systems to conserve and enhance biodiversity through the following: implementation of relevant laws and policies, agreeing to harmonized indicators to measure progress, allocating and funding monitoring bodies, and creating a system of robust and transparent reporting and use of enforcement mechanisms. The full list of proposed actions (including research, innovation, policy, and management) is available in table S2.

We undertook document analysis to examine the extent to which the eight key actions were addressed by the following policy frameworks: the Aichi Targets (*9*), the SDGs (*44*), the CBD Zero Draft of the Post-2020 Global Biodiversity Framework (*20*), and the updated Zero Draft (*45*). As this is a changing area of policy, our analyses represent a snapshot of the current situation. From this analysis, we identified synergies and tensions among targets, as well as gaps (summarized in Table 2, listed fully in table S3). From a further literature review, we identified the barriers and opportunities in implementing the key actions (table S4), acknowledging the integrated nature of food systems, at the global level and in relation to two country case studies: Peru and the United Kingdom (*Box 1*). These countries have different contexts in terms of biodiversity, and food production and consumption patterns, which allowed us to consider the feasibility of the proposed actions. It also allowed us to investigate global-local interactions; in particular, whether “global” policies are sensitive to heterogeneous local conditions and/or particular societal groups, considering the barriers and conditions for the proposed actions to support transformative change, acknowledging that food systems are teleconnected across scales, nested, and interact with future (unforeseen and uncertain) changes. From the analysis of the actions from the science-policy fora at the global level, and in relation to Peru and the United Kingdom, the authors considered how targets and indicators corresponding to each key action could be developed into Specific, Measurable, Achievable, Relevant and Time-bound (SMART) indicators to support transformative change in food systems (*46*). We took into account existing indicators applicable worldwide and for which data and methodologies exist, as well as information gaps, where further research is needed. On the basis of the barriers and opportunities identified, we considered the enabling conditions needed to effectively achieve the proposed targets and broader actions required by societal actors to support sustainable food production and consumption in the post-2020 biodiversity framework (Fig. 1).

Box 1 **Peru** is one of the world’s megadiverse countries, still retaining vast natural ecosystems (over 60% of which are natural forests) with low human intervention. Of its 1,280,000-km^2^ land area (*47*), agriculture makes up 18.5% (*48*). Peru has a population of 32.5 million (*49*), with a carbon footprint of 1.86 metric tons per capita (*50*). Peru’s top commodities produced (in metric tons) are sugar cane, potatoes and rice, and its top export commodities are avocados, grapes, and coffee (*51*). Peru’s fisheries are of global importance, and the country is the leading world exporter (and producer) of fishmeal and fish oil (*52*). Peru is a center of origin for diverse food crops of global importance; for which its domestic agrobiodiversity, wild relatives, and associated traditional knowledge constitute an important part of its natural capital (which has to date not been measured). The World Bank recognizes Peru as one of the emerging economies in Latin America (*53*). A member of the CBD since the beginning, Peru established a National Commission, consisting of a range of government sectors, civil society, and indigenous people. The Ministry of Environment oversees biodiversity policy, and each of the 24 regional governments has developed a regional biodiversity strategy. In future projections of biodiversity loss—in scenarios where drivers of change do not deviate from the current socioeconomic and governance trajectory—tropical regions face particular combined risks of declines due to the interactions between climate change, land-use change, and fisheries exploitation (*8*).

**The United Kingdom.** Agricultural land makes up 71.7% (*48*) of the total land area of the United Kingdom’s total [241,930 km^2^ (*47*)]. Its population of 66.8 million (*49*) has a carbon footprint of 5.78 metric tons per capita (*50*). The United Kingdom’s top three commodities produced (in metric tons) are cow’s milk, wheat, and sugar beet, with top export commodities being distilled alcoholic beverages, milk, and barley (*51*). In terms of governance of environmental matters and sustainable development, England and the three devolved administrations (Wales, Scotland, and Northern Ireland) are responsible for their own legal and policy responses. Each has a different biodiversity strategy. The process of the United Kingdom leaving the European Union (EU) presents substantial uncertainties for actions on sustainable food production and consumption. The EU, itself a signatory of the CBD and committed to the SDGs, had harmonized the approaches taken within the United Kingdom to some extent to fit within the framework set by EU law and policy. On withdrawal from the EU, each devolved administration will have more freedom to develop and vary their legal and policy responses relating to sustainable production and consumption, leading to potentially divergent approaches within the United Kingdom. The United Kingdom as a whole will continue as a member of the CBD.

## TARGETS AND INDICATORS FOR SUSTAINABLE FOOD PRODUCTION AND CONSUMPTION

From our analysis, we propose targets and associated actions identified in the science-policy fora (Table 1). In combination, these could be explicitly incorporated into the CBD’s post-2020 biodiversity framework (see associated targets proposed in the updated Zero Draft in Table 1) to help meet the aim of sustainably obtaining sufficient food for people, while conserving and restoring biodiversity by stopping habitat loss from agriculture and unsustainable fishing practices, and thus support the CBD’s strategic objective for sustainable use of biodiversity. We suggest a progressive timeline for targets up to 2030, following the frequency of CBD COPs, but these targets should not preclude taking action in shorter periods. For these targets to be effective and meaningful, wider enabling conditions including more equitable distribution and responsibilities for implementation by diverse societal actors must be considered.

**Table 1:** Key actions from science-policy fora and their proposed targets and indicators. OECD, Organisation for Economic Cooperation and Development.

**Action**	**CBD Updated Zero** **Draft target**	**Proposed targets**	**Proposed indicators**
1. Remove incentives that make food production and consumption harmful to biodiversity	E. 12. (c) 17.	By 2025, parties identify incentives harmful to biodiversity.	Number of countries with policy plans for removal or reform of incentives harmful to biodiversity.
			Percentage of harmful subsidies removed and/or redirected (e.g., at least 50% by 2030, 100% for 2050).
		By 2025, develop policy plans, including a prioritized list of measures, with timelines, leading to the eventual removal, phase-out, or reform of incentives harmful to biodiversity	Sector-level government financial transfers to agriculture [Organisation for Economic Co-operation and Development (OECD) databases].
		By 2025, redirect capacity-enhancing subsidies (subsidy programs that lead to disinvestments in natural capital assets once the fishing capacity develops to a point where resource exploitation exceeds the Maximum Economic Yield) to support sustainable activities.	Proportion of assessed fish stocks that are overfished [Food and Agriculture Organization of the United Nations (FAO) statistics].
2. Accounting for true value and true costs of production by sector	E. 12. (c) 13.	By 2025, a system of natural capital accounting is developed including economic, cultural, social, intrinsic, and intergenerational values of biodiversity, including diverse conceptualization of multiple values of nature.	Frequency of use of valuation tools that assess the diverse conceptualization of multiple values of nature and its benefits.
			Number of countries that have developed natural capital accounting systems in their National Development Plans, which take into account the explicit role of nature into poverty reduction strategies and other key development plans, by including economic, cultural, social, intrinsic, and intergenerational values of biodiversity.
3. Reduce food waste and loss across supply chains	Not explicitly mentioned. Other relevant targets: E. 12. (b) 9, E. 12. (c) 14, E. 12. (c) 15.	By 2030, halve per capita global food waste at the retail and consumer levels and reduce food losses along production and supply chains, including postharvest losses.	Use of Accounting and Reporting Standard (Food Loss and Waste Protocol Standard).
			Number of countries reporting to Global Food Loss Index and National Food Loss Index.
4. Strengthen sustainability standards and certification	Not explicitly mentioned. relevant targets: E. 12. (b) 9, E. 12. (c) 14, E. 12. (c) 15.	By 2025, sustainability certification standards strengthen biodiversity requirements, including No Net Loss as a minimum and management and monitoring of conservation areas (e.g., areas of High Conservation Value as specified in standards).	Number of companies with biodiversity commitments/policies and their market share.
		By 2025, producing governments require minimum sustainability standard for export.	Number of companies reporting against SMART biodiversity indicators.
		By 2025, consuming countries require sustainability certification for import of high-biodiversity risk commodities.	% of ISEAL Alliance members with stronger biodiversity requirements, including No Net Loss as a minimum, and management and monitoring of conservation areas (e.g., High Conservation Value areas).
		By 2025, sustainable public procurement plans adopted by governments.	
		By 2025, sustainability certification standards include recognition of need for enhancing agrobiodiversity, land sharing, and multifunctionality.	Use of Biodiversity Impact Indicators for Commodity Production (BIICP).
		By 2025, standards include recognition and autonomous rights of indigenous people and local communities.	
5. Promote the use of life cycle assessments	Not explicitly mentioned. Other relevant targets: E. 12. (b) 9; E. 12. (c) 14; E. 12. (c) 15.	By 2025, Life Cycle Assessment and ecological footprints are made freely available to the consumer when buying a product.	Ecological footprint across life cycle of product.
		By 2025, data are aggregated and monitored at municipal/ national levels using standardized protocols.	
		By 2025, Life Cycle Assessment reporting includes multiple stakeholders, e.g., small-scale farmers and informal markets.	Number of products with life cycle assessments.
		By 2025, waste management is tracked and disclosed at all levels of Life Cycle Assessments.	
6. Promote sustainable and varied diets	Not explicitly mentioned. Relevant targets: E. 12. (b) 8, E. 12. (b) 9; E. 12. (c) 15.	By 2025, develop dietary guidelines that address health and environmental sustainability, promoting a more diverse and nutritionally balanced diet of fruits, vegetables, meat, and seafood.	Number of countries with dietary guidelines that address both health and sustainability.
			Meat consumption kilograms per capita.
			Seafood consumption kilograms/capita (FAO statistics).
		By 2025, develop incentives for redirecting reduction fisheries (i.e., fisheries, often on lower trophic levels, that process their catch into fish meal or fish oil) to direct human consumption.	Quantities of reduction fisheries (FAO statistics).
		By 2025, develop incentives for increased mariculture of edible sea plants and filter feeders.	Quantities of maricultured sea plants and filter feeders (FAO statistics).
		Financial incentives for environmentally friendly and healthy food production and consumption.	SDG 12.1.1 Number of countries with sustainable consumption and production (SCP) national action plans or SCP mainstreamed as a priority or a target into national policies.
		By 2025, schools provide sustainable varied meals to children up to the age of 12, following the dietary guidelines.	SDG 2.1.2 Prevalence of moderate or severe food insecurity in the population, based on the Food Insecurity Experience Scale (FIES).
7. Mainstream biodiversity considerations in food systems (cross-cutting)	E. 12. (c) 13, E. 12. (c) 14, E. 12. (c) 15, E. 12. (c) 16; E. 12. (c) 17; E. 12. (c) 18; E. 12. (c) 19, E. 12. (c) 20.	Cross-cutting action: Implementation of actions contributes to mainstreaming biodiversity (Fig. 1). Includes integrating biodiversity into national and local planning, development processes, and poverty reduction and accounts.	
8. Strengthen governance of sustainable food production and consumption (cross-cutting)	G. 14. (a), G. 14. (b), G. 14. (g), H. 15, H. 16, H. 17, H. 18.	Cross-cutting action: Strengthening governance within and beyond the CBD contributes to the implementation of actions and creates “enabling conditions” for effectiveness (Fig. 1). Undertaken through implementation of relevant laws and policies, agreeing to harmonized indicators to measure progress, allocating and funding monitoring bodies, and creating a system of robust and transparent reporting and use of enforcement mechanisms.	

## BARRIERS AND OPPORTUNITIES FOR IMPLEMENTING ACTIONS

Food production and consumption are addressed in the Aichi Targets, the SDGs, the CBD Zero Draft, and the updated Zero Draft document (Table 2), but synergies, tensions, and gaps exist. The differing ambitions of the Aichi Targets and the SDGs, in relation to biodiversity conservation and sustainable use, creates tensions because states could prioritize the SDGs related to economic development over sustainability and biodiversity conservation, thus missing how nature ultimately underpins most of the SDGs and is fundamental to human well-being (*54*). Although the 2030 Agenda for Sustainable Development provides a major opportunity for tackling indirect drivers of biodiversity loss, there is limited consideration of the impacts on biodiversity or the role biodiversity plays when addressing food production and consumption (*55*, *56*). Nor does it consider biodiversity-related feedbacks leading to lock-ins that limit progress toward the SDGs (*57*).

**Table 2:** Summary of actions related to sustainable food production and consumption in the SDGs, Aichi Targets, the CBD Zero Draft, and the CBD Updated Zero Draft. Full policy wording in table S3.

**Actions**	**SDGs**	**Aichi Targets**	**CBD Zero Draft**	**CBD Updated Zero Draft**
1. Remove incentives that make food production and consumption harmful to biodiversity	2.b, 14.6.	3	D. 12. (c) 12.	E. 12. (c) 17.
2. Accounting for true value and true costs of production by sector	15.9	2	D. 12. (c) 13.	E. 12. (c) 13.
3. Reduce food waste and loss across supply chains	12.3	Not explicitly mentioned. Other relevant target: 4.	Not explicitly mentioned. Other relevant targets: D. 12. (b) 8, D. 12. (c) 14, D. 12. (c) 17.	Not explicitly mentioned. Other relevant targets: E. 12. (b) 9, E. 12. (c) 14, E 12. (c) 15.
4. Strengthen sustainability standards and certification	Not explicitly mentioned. Other relevant targets: 2.4, 12.6, 14.4.	Not explicitly mentioned. Other relevant targets: 4, 6, 7	Not explicitly mentioned. Other relevant targets: D. 12 (b) 8, D. 12 (c) 14, D. 12 (c) 17.	Not explicitly mentioned. Other relevant targets: E. 12. (b) 9, E. 12. (c) 14, E. 12. (c) 15.
5. Promote the use of life cycle assessments	Not explicitly mentioned. Other relevant targets: 2.4, 8.4.	Not explicitly mentioned. Other relevant target: 4.	Not explicitly mentioned. Other relevant targets: D. 12. (c) 14; D. 12. (c) 17.	Not explicitly mentioned. Other relevant targets: E. 12. (b) 9; E. 12. (c) 14; E. 12. (c) 15.
6. Promote sustainable and varied diets	Not explicitly mentioned. Other relevant targets: 2.4, 14.4.	Not explicitly mentioned. Other relevant targets: 6, 7.	Not explicitly mentioned. Relevant target: D. 12. (c) 17.	Not explicitly mentioned. Relevant targets: E. 12. (b) 8, E. 12. (b) 9; E. 12. (c) 15.
7. Mainstream biodiversity considerations in food systems (cross-cutting)	8.4	4, 19.	I. 8. (i), D. 12. (c) 12, D. 12. (c) 13, D. 12. (c) 14, D. 12. (c) 15, D. 12. (c) 16, D. 12. (c) 17, D. 12. (c) 18, D. 12. (c) 19, D. 12. (c) 20.	E. 12. (c) 13, E. 12. (c) 14, E. 12. (c) 15, E. 12. (c) 16; E. 12. (c) 17; E. 12. (c) 18; E. 12. (c) 19, E. 12. (c) 20.
8. Strengthen governance of sustainable food production and consumption (cross-cutting)	2.4, 8.4, 9.4, 12.2, 16.6, 16.7, 16.8.	4, 7, 13, 17, 18.	8. (d), 8. (f), 8. (g), F. 14. (g), G. 16. (a), G. 16. (b), Annex I I. B. 3.	G. 14. (a), G. 14. (b), G. 14. (g), H. 15, H. 16, H. 17, H. 18.

We found that a range of multiscalar challenges and opportunities exist in implementation of the eight actions. We discuss these in relation to the global level policy analysis and in relation to the two country case studies, where applicable. We note that any actions taken within the two country case studies will likely have positive and negative impacts beyond their boundaries through telecoupling and in response to future changes. In relation to the actions, we discuss the implications of these barriers and opportunities for deriving targets and indicators for the CBD’s post-2020 biodiversity framework (as presented in Table 1). The reviews of implementation at the global level and at the level of the two country case studies reveal several contextual and cross-cutting political, economic, social and technical challenges (Table 3). From this analysis, we identify the necessary enabling conditions through which the post-2020 biodiversity framework can support transformative change in food systems, informed by the country case studies and global-level review (Table 4).

**Table 3:** Cross-cutting challenges and enabling conditions in implementing actions for the sustainable production and consumption of food. NGOs, nongovernment organizations.

**Challenges**	**Enabling conditions to overcome challenges**	**Supporting key actions**
Existing economic development trajectories, including “agriculture for development” through large-scale high-input farming.	Consideration of more diverse and equitable development pathways including consideration of biodiversity in food production systems and development projects (ecological intensification, agroecology).	1, 7, 8
	Synergies with other global sustainability agendas.	7, 8
	Focusing on accountability of key (and sometimes less visible) industries in demanding sustainable change (e.g., commodity traders).	7, 8
	Clear standards and protocols for reporting against targets on biodiversity and sustainable production and consumption, to be developed and used by all actors and stakeholders in the production and consumption chain.	3, 4, 5, 7, 8
	Gathering more data and establishing harmonized indicators to measure effectiveness and track progress of policies on sustainable consumption and production and links with biodiversity.	4, 7, 8
Lack of/weak regulation of unsustainable production and consumption.	Political will and integrative governance.	7, 8
Lack of a unified food system perspective using important complementarities of agriculture, fishery, and aquaculture to optimize nutritional value and biodiversity.	Progressive regulation by governments to support more sustainable production and consumption (i.e., national strategies and action plans for sustainable consumption and production) to enhance the power of environmental norms.	8
Conflicting objectives between stakeholders (e.g., nongovernmental organizations and companies) and within stakeholder groups (e.g., between government departments).	Taxation and levies to support biodiversity monitoring and research and pro-poor objectives in food supply chains; incorporating and supporting Life Cycle Assessment and standards.	4, 5, 7, 8
Lack of compliance by governments against CBD requirements related to food production and consumption.	Setting specific goals to national contexts, matching global targets; more effective compliance mechanisms within and beyond the CBD through greater accountability for industry and government practices.	7, 8
Strong resistance from corporate actors and lack of accountability for private sector and effects on biodiversity; industry lobbying and political power maintains business as usual.	Greater engagement and inclusive processes in CBD by agents beyond conservation professionals, including policy-makers and practitioners in economic, industry, and trade sectors.	7, 8
Lack of transparency of trade agreements, supply chains, and commodity prices.	Partnerships, businesses demonstrating leadership through use of science-based equitable commitments (including to “no net loss” and restoration activities), strengthening accountability, compliance, transparency, Life Cycle Assessment, and standards.	4, 5, 6, 7, 8
	Progressive laws and regulations to hold private sector to account (including in no net loss and restoration activities).	8
Uncertainties/complexity in understanding the direct and indirect impacts of food production and consumption patterns.	Mutual learning and support: connecting science and policy actors, indigenous and local knowledge; appreciating and exchanging respective multidisciplinary and transdisciplinary knowledges.	7, 8
Interdisciplinary scientific and local/indigenous knowledge undervalued.	Change in behavior at all levels (governments, business, producers, and consumers).	7, 8
Sociocultural factors and perceptions of individual rights, e.g., increasing meat consumption globally; inequality and uneven consumption patterns; and lack of consideration of food waste.	Shifts in individuals’ perspectives, including appreciation of diverse conceptualizations of links between food and nature through community education activities.	7, 8
	Learning how diverse and alternative visions and narratives of sustainability consider trade-offs and outcomes in relation to sustainable production and consumption of food.	7, 8

**Table 4:** Agents and actions for change to create enabling conditions for transformative changes in food production and consumption for the post-2020 biodiversity framework.

**Agents**	**Actions for change**	**Key actions**
Small-/medium-scale farmers	Diversification of production activities; recognizing importance of biodiversity; collective action with other farmers, including to establish wildlife corridors with other land users; and engagement with standards and ecological intensification	3, 4
Large-scale producers	Diversification of production activities; integrating values/costs of biodiversity; science- based commitments and targets and transparent reporting on progress (including to no net loss and restoration activities); promote agrobiodiversity, ecological intensification, agroecology; compliance with sustainability standards and legal requirements; and scrutiny over transactions including “publish what you pay” for agribusiness	2, 3, 4, 5, 7
Citizens	Awareness of biodiversity impacts in supply chains; shifts in perceptions and behavior (reduced consumption of unsustainable foods, diet); social learning; citizens assemblies; hold industry and government to account; citizens assemblies; local green politics; urban farming	3, 6, 8
Local communities and indigenous peoples	Hold industry and government to account; citizens assemblies; local green politics; urban farming; and value and maintain local and traditional knowledge related to food	2, 4, 8
Local/regional governments	Hold industry to account; sustainable procurement; taxation; awareness campaigns; and stronger anti-corruption measures	7, 8
Non-governmental organizations/ Civil society organizations	Holding governments and industry to account to recognize and address biodiversity loss and links with production and consumption of food; education of consumers; supporting activist groups; strengthening standards; and strict requirements for engaging with business	1, 2, 3, 4, 5, 6, 7, 8
Businesses	Legal compliance; companies adopt doughnut economics model; science-based commitments (including to no net loss and restoration activities); companies held to account and able to demonstrate compliance with regulations and standards; transparency of reporting; resources dedicated to implementation of strong commitments including social aspects and meaningful engagement with diverse range of stakeholders; financing independent legal support where needed; internalizing costs of monitoring; sustainable procurement; and diverse business models including social enterprises and cooperatives	1, 2, 3, 4, 5, 6, 7, 8
Consultants/Experts	Greater independence and codes of conduct on representation of private interests; peer review; and integrating local and traditional knowledge	2, 3
Governments	Monitoring; review current incentive programs; enforcement of regulations; support to low-income groups for sustainable healthy diets; stronger controls of advertising encouraging unsustainable product purchases; taxation/levies; supporting alternative development pathways: GDP alternatives (incorporation of quality of life/well-being/ just sustainability); anticorruption measures; delivering awareness campaigns to citizens and businesses; develop and democratize natural capital accounting systems that incorporate noneconomic values; regulate companies to reduce and report on food loss and waste reduction; and require, develop and support standards for sustainable production and consumption	1, 2, 3, 4, 5, 6, 7, 8
Standards bodies	Strengthen compliance and assurance mechanisms of standards; introducing stronger biodiversity aspects in standards; strengthen transparency measures; shift from single commodity certification to valuing diverse landscape use and agroecology; and valuing diverse perspective and knowledges	3, 4, 8
Research communities	Exchanging multidisciplinary knowledge with policy communities; valuing diverse perspective and knowledges; supporting social and technological innovation; and attention to justice and equity concerns, capacity building, methodologies for accountability including in no net loss and restoration activities	2, 5, 6, 8
Funding agencies	Consistently including biodiversity concerns in financing decisions; use of mitigation hierarchy (for limiting as far as possible the negative impacts on biodiversity from development projects) including clear “no development” option if biodiversity loss too great; considerations of funding habitat restoration; and microcredit schemes for biodiversity	7, 8
Private investors	Engagement with biodiversity issues and sustainable production and consumption; incorporating strong environmental, social and governance (ESG) criteria into screening processes; divestment from most harmful industries; promotion of or engagement in development and inclusion of biodiversity driven standards along the supply chain, Life Cycle Assessment; and invest in income-sensitive, efficient storage technologies	5, 7, 8

### Action 1. Remove incentives that make food production and consumption harmful to biodiversity

#### Global level policy findings

This action is partly addressed by the SDGs (6, 2.b, 2.b.1), Aichi Target 3, and the CBD Zero Draft [D. 12 (c) 12] and its update [E. 12 (c) 17]. The removal, phase-out, or reform of harmful incentives are part of the Aichi Targets and SDG 6 but are insufficiently addressed in associated indicators, thus highlighting ambiguity in the concrete actions needed and a lack of accountability for action. Politically, incentives are often difficult to reform because of strong opposition from recipients and tight linkages with regional and international trade. Shifts in subsidies may have negative economic impacts on low income and poorly resourced producers if they are insufficiently thought out (*58*).

#### Barriers and opportunities for implementation in Peru

In Peru, development pathways based on agricultural expansion supported by credit policies can be harmful to biodiversity (*59*). These incentives are complex and deeply connected to national goals of economic growth and territorial control (*60*, *61*) and international trade agreements, e.g., U.S.-Peru Trade Promotion Agreement. While there are no official “subsidies” to agriculture, the 2001 Agrarian Promotion Law has allowed the payment of less income tax and a more flexible labor regime for the agricultural sector (which has been reflected in the growth of agribusiness). An extension of the Agrarian Promotion Law will also benefit the aquaculture and forestry sectors. There could be a hidden subsidy for nontraditional exporters (including agribusinesses), whereby returns of tariffs paid for importing inputs has been reduced from 4% before 2019 to 3%. Thus, both “official” and unofficial (or direct/indirect) subsidies must be considered.

#### Barriers and opportunities for implementation in the United Kingdom

With the United Kingdom (U.K.) leaving the EU, there may be shifts in political and trade barriers, as well as uncertainties for U.K. food production following its withdrawal from the EU Common Agricultural Policy (CAP). While the CAP was created to ensure food security and economic viability for rural farming communities following World War Two, the subsidizing of food production has substantially affected natural habitats, while driving over-production of various commodities (*62*). Post-CAP U.K. policy states that there will be a move toward future generations of farmers supported in restoring natural habitats as part of an explicit proenvironmental agenda (*63*), but some environmental groups have expressed concern for potentially weaker regulations in relation to U.K. pesticide use (*64*, *65*). Attention to these concerns is required by a range of actors to ensure accountability.

#### Implications for targets and indicators

Incentives harmful to biodiversity are often difficult to identify as effects on biodiversity may be indirect, diverse, and context specific (*66*), so we propose an initial step related to their clear identification (by 2025, parties identify incentives harmful to biodiversity). In many cases, this will require cooperation between states, and research is needed to compile data to support this indicator. Given the difficulties mentioned, we suggest that governments support this action by compiling a list of measures, with timelines, leading to the eventual removal, phase-out, or reform of incentives harmful to biodiversity by 2025. An optional target could be that by 2025, subsidies are redirected to support sustainable activities, e.g., subsidizing actions that reduce or hamper investments in fish exploitation assets once exploitation of a fishery exceeds its Maximum Economic Yield. Indicators might include the number of countries with policy plans for removal or reform of incentives harmful to biodiversity, percentage of harmful subsidies removed and/or redirected, and metrics for tracking progress toward attaining this target, such as sector-level government financial transfers to agriculture or the proportion of assessed fish stocks that are overfished.

### Action 2. Accounting for true value and true costs of production by sector

#### Global level policy findings

The SDGs and the CBD recommend integrating biodiversity values into national and local planning, development processes, and poverty reduction strategies and accounts [SDG 15.9, Aichi Target 2, CBD Zero Draft E. 12. (c) 13], indicating some alignment on policy language. However, details on how these should be integrated, beyond strategic environmental assessments and environmental impact assessments, are lacking. Governmental policies and market transactions typically do not reflect the full value of nature’s contributions to people (*67*). An important barrier to effective implementation of natural capital accounting is the current lack of interdisciplinary and transdisciplinary competences to support integration of knowledge systems from indigenous and local people into scientific analysis and policy-making (*68*). Currently, nonmonetary values that are not amenable to economic methods including other worldviews and associated values are rarely considered, including those associated with individual and shared sociocultural values, those underpinned by indigenous local knowledge, as well as other biophysical and health-related values (*68*).

#### Barriers and opportunities for implementation in Peru

Considering the potential for natural capital accounting in Peru, implementation is still in its infancy. Peru’s National Strategy for Biological Diversity for 2021 Action Plan 2014–2018 states that “by 2018 two ecosystem services should have been valued, ensuring ecosystem integrity and respect for the indigenous peoples involved” (*69*). The World Bank–led “Wealth Accounting and the Valuation of Ecosystem Services” global partnership piloted a project from 2009 to 2013 in the Department of San Martin, Peru, which argued that for natural capital accounting to be fully used, it needs to be integrated into national information systems and continuously measured (*70*).

#### Barriers and opportunities for implementation in the United Kingdom

The United Kingdom has made a commitment to natural capital accounting and set up a working group, to develop a methodology to drive biodiversity benefits and climate mitigation (*71*). It has been argued that the actual measurement of natural capital is difficult because of the lack of a baseline, and substantial progress toward the targets has been lacking (*72*). However, an example of positive progress is seen in Scotland, which has developed a progressive Natural Capital Asset Index, which does not include monetary values but is composed in a way that reflects the relative contribution of natural habitats to human well-being (*73*). Work on natural capital accounting, up to now, may not take into account that food production and consumption at one location sometimes lead to impacts on the environment and people at distant locations (*74*).

#### Implications for targets and indicators

Because of various technical and other drawbacks, natural capital accounting may not be ready for mainstream use. Rather, we propose that alternative forms of valuation are further developed (Table 3). As a target, we propose that by 2025, a system of natural capital accounting is developed, which includes economic, cultural, social, intrinsic, and intergenerational values of biodiversity. Indicators could include the number of countries that have developed natural capital accounting systems in their National Development Plans, which take into account the explicit role of nature into poverty reduction strategies and other key development plans, by including economic, cultural, social, intrinsic, and intergenerational values of biodiversity. Progress could also be tracked by measuring the frequency of use of valuation tools that assess the diverse conceptualization of multiple values of nature and its benefits.

### Action 3. Reduce food waste and loss across supply chains

#### Global level policy findings

Focusing on productivity and efficiency, the SDGs contain targets on food waste at the retail and consumer levels, as well as aiming to reduce food losses along production and supply chains (*44*). Food waste was not mentioned in the Aichi Targets or CBD Zero Draft documents, but proposed targets refer to reducing productivity gaps [E. 12. (b) 9] and ensuring that production practices and supply chains are sustainable [E. 12. (c) 14]. Food waste and loss are context dependent and linked with development pathways. Rapid urbanization and globalization mean that food supply chains require adequate roads, transportation, and marketing infrastructure (*75*). Addressing food waste is currently challenging because of shifts toward items with short shelf life (*75*).

#### Barriers and opportunities for implementation in Peru

In Latin American and the Caribbean, food loss at the retail stage is estimated at 220 million metric tons (*76*). In Peru, an estimated 2.5 million people suffer from hunger, with 33% of the food produced going to waste (*77*). In 2019, Peru passed a law (Law No. 30988), sponsored by the Ministry of Agriculture, to design and implement strategies to improve the efficiency of the food supply chain, from primary production to human consumption (*78*).

#### Barriers and opportunities for implementation in the United Kingdom

The United Kingdom’s Courtauld Commitment requires food supply chain companies to cut food waste by 20% by 2025 (*79*), and the 25 Year Environment Plan calls for a 20% cut in food waste per capita by 2025, requiring a further 30% cut to meet the SDG target of halving per capita global food waste at retail and consumer levels by 2030 (*63*). However, relatively low food prices in the United Kingdom and retailers encouraging overspending may contribute to food waste. Furthermore, using food waste to produce commercially sold fuel gas may discourage food waste reduction (*80*).

#### Implications for targets and indicators

We propose that the post-2020 biodiversity framework endorses the SDG target on global food waste (12.3), which would strengthen its implementation. This is likely to require changes in objectives and behavior of businesses and consumers, as well as new progressive laws and regulations. The CBD could therefore include a target that aligns with the SDG target 12.3, which states that “by 2030, halve per capita global food waste at the retail and consumer levels and reduce food losses along production and supply chains, including post-harvest losses” (*44*). A potential indicator could include the number of countries reporting a National Food Loss Index, and the Food Loss and Waste Accounting and Reporting Standard could be used to support in measuring progress (*81*).

### Action 4. Strengthen sustainability standards and certification

#### Global level policy findings

Sustainability standards and certification promote sustainable production practices from a distance (*82*). However, their use is not explicitly mentioned in the SDGs, Aichi Targets, or CBD Zero Drafts. Despite some progress in developing and tightening private voluntary sustainability standards (*83*, *84*), there is only limited evidence for the potential for standards to support in bending the curve on biodiversity loss (*85*). Standards for sustainable agriculture contain limited biodiversity guidelines (*86*). Although Marine Stewardship Council–certified seafood has been found to be three to five times less likely to be subject to harmful fishing than uncertified seafood (*87*), certification requirements have found to be too lenient and ambiguous (*88*). We suggest that the biodiversity aspects of standards are strengthened. Currently, sustainability standards focus on a small number of agricultural commodities, and a small proportion of total farmland is covered by certification schemes (*89*). Although sustainability standards are designed to incentivize sustainable production practices through demand for certified products, there are concerns that certified production may outweigh market demand (*89*, *90*).

#### Barriers and opportunities for implementation in Peru

Peru is one of the top five standard compliant cocoa producers (*90*) and accounts for 25% of certified seafood production (*91*). Peruvian cocoa is mostly exported to Europe (*92*), so consistent demand for sustainable products could provide a market driver for more sustainable production. In a context of “land grabbing” (*93*), the effects of standards in preventing biodiversity loss may be severely limited unless broader complex structural conditions are addressed. Allocations of land rights, high labor and input costs, persistence of pests, and unstable cocoa yields in the context of climate change are issues faced by many farmers that cannot be addressed adequately by certification and standards (*94*).

#### Barriers and opportunities for implementation in the United Kingdom

In the United Kingdom, national supermarkets have an important role in food spending (77% of all main shopping trips) (*95*) and can make strong demands of their suppliers on sustainability through the use of standards (*96*). Use of these standards by regulators, public procurement policies, and import and export taxes to support sustainable trade could support fairer contracts and more sustainable and biodiversity-friendly practices across global value chains (*96*).

#### Implications for targets and indicators

Given the need to strengthen biodiversity requirements in sustainability certification, we suggest that by 2025, No Net Loss is incorporated into standards as a minimum and that long-term management and monitoring is implemented for conservation areas (e.g., with “High Conservation Value,” as used in certification standards). Standards should include recognition and autonomous rights of indigenous people and local communities. Because of the risks of standards creating exclusions and large-scale bias, they should also incorporate agrobiodiversity and multifunctionality. Producing and consuming countries can also provide more support for the uptake of standards, with producing countries requiring minimum sustainability standard for export and consuming countries requiring sustainability certification for import of high-biodiversity risk commodities. Governments’ public procurement plans should also incorporate sustainability requirements. Research into the effectiveness of integrated landscape approaches in supporting biodiversity and sustainable development goals, and the role of sustainability certification in these, is an important frontier for research. Potential indicators could include the number of companies using SMART biodiversity indicators and the percentage of ISEAL Alliance (International Social and Environmental Accreditation and Labelling Alliance) members (sustainability standards) with stronger biodiversity requirements, including “No Net Loss” as a minimum and management and monitoring of High Conservation Value areas. Biodiversity Impact Indicators for Commodity Production could also support in strengthening the impacts of standards.

### Action 5. Promote the use of life cycle assessments

#### Global level policy findings

Life Cycle Assessment is the most widely used method to assess environmental impacts of agricultural products over their full life cycles (*97*), from planting and fertilization to consumer packaging and waste, and can be used to support environmental policies (*98*). SDG 8.4 states a target to “Improve progressively, through 2030, global resource efficiency in consumption and production and endeavour to decouple economic growth from environmental degradation, in accordance with the 10 Year Framework of Programmes on Sustainable Consumption and Production, with developed countries taking the lead,” but this process of decoupling remains elusive. SDG 12 states the need to achieve environmentally sound management of chemical and wastes in air, soil, and water throughout the life cycle of production-consumption (12.4), but Life Cycle Assessment is not explicitly mentioned as a possible tool to support achieving this. Although the Aichi Targets and Draft Zero documents do not mention Life Cycle Assessment, related targets include requirements for sustainable production and consumption plans (Aichi Target 4), supporting the productivity, sustainability, and resilience of biodiversity in agricultural and other managed ecosystems [E. 12. (b) 9], ensuring sustainable production practices and supply chains [E. 12. (c) 14] and sustainable consumption [E. 12. (c) 15] (*45*). There are complexities in capturing all impacts in current Life Cycle Assessment methodologies, including what constitutes a product’s “biodiversity footprint” (*99*, *100*) and how much of an adjacent or indirectly connected area is affected. These methodologies often lack the spatial resolution and predictive ecological information to reveal key impacts on climate, water, and biodiversity (*101*), and current methodologies tend to favor high-input intensive agricultural systems and misrepresent less intensive or smaller-scale agroecological systems (*97*). In sum, while Life Cycle Assessment and the “life cycle” point of view are pertinent to reforms of the CBD, they have certain drawbacks that should be addressed if they are to become part of global policy efforts to protect biodiversity.

#### Barriers and opportunities for implementation in Peru

Life cycle assessments of the fishing industry usually focus on main fishing activities. Nevertheless, a study of 136 vessels of the Peruvian industrial anchoveta (anchovy) fleet found that significant environmental impacts stem from other parts of the industry, in particular, construction of the fleet (∼11%) and its maintenance (∼23%) (*102*). This leads to an underestimation of life cycle impacts. Peru has recently opened a Centre for Life Cycle Analysis as part of the Life Cycle Initiative, supported by the United Nations Environment Programme (*103*), which presents an opportunity to improve understanding of biodiversity impacts associated with products’ life cycles in Peru.

#### Barriers and opportunities for implementation in the United Kingdom

Apart from the uncertainties of Life Cycle Assessment methodologies noted above, additional uncertainties arise from telecoupled impacts. For example, assessments of cattle production systems take into account on-farm management data but may not consider the impacts occurring at the source of cattle feed (*104*). This is important in the United Kingdom given its focus on meat production and consumption and its embedded biodiversity footprint. Temporal variations in environmental impacts have been observed, for example, in Life Cycle Assessment studies examining lettuce and raspberries consumed in the United Kingdom, because of differences in yields related to variable weather conditions (*105*, *106*).

#### Implications for targets and indicators

We propose that consumers be given greater access to information about life cycle impacts and ecological footprints of the products that they purchase (e.g., by 2025). This would help consumers to better understand the impacts of products on biodiversity, while mainstreaming biodiversity concerns of food consumers. Associated indicators could include the ecological footprint across the life cycle of product and the availability of information about life cycle impacts for a particular product. An important challenge for the implementation of Life Cycle Assessment is to address transparency of agricultural products and fisheries in opaque supply chains. There are also important questions related to responsibility for data collection and who monitors, evaluates, and audits, as this could pose burdens for data collection needs and management. Given the problems associated with current methodologies, the development of standardized protocols by 2025 would be beneficial, with data aggregated and monitored at municipal/national levels. As current methodologies misrepresent less intensive or smaller-scale agroecological systems, the development of Life Cycle Assessment reporting should include multiple stakeholders, e.g., small-scale farmers and informal markets, to address the challenge of exclusion of certain actors and development pathways.

### Action 6. Promote sustainable and varied diets

#### Global level policy findings

While the SDGs clearly seek to address issues of nutrition, “sustainable diets” are not mentioned. The Aichi Targets 6 and 7 mentioned the goals of sustainable agriculture and fishing but did not explicitly mention diets. The CBD Draft Zero documents state that “People everywhere take measurable steps towards sustainable consumption and lifestyles, taking into account individual and national cultural and socioeconomic conditions, achieving by 2030 just and sustainable consumption levels.” This emphasizes the importance of food security and inequality in distribution and access (*107*) and acknowledges that diets are embedded in cultural, social, and ecological contexts and that food and eating practices are specific to people and places (*108*). However, the flexible language proposed shows a lack of clarity on who is responsible to take these “measurable steps,” and precisely how.

Taking the example of animal product consumption, it is understood that shifting toward more sustainable and varied diets that include fewer animal products could support people (particularly in the global North) in reducing their high environmental footprints (*109*). However, there are numerous political and economic barriers to doing so. These include the powerful meat and dairy industries (*110*, *111*), subsidies supporting unsustainable production and consumption (*112*), and a lack of uptake of the issue by environmental groups (*112*, *113*). Technically, there are complexities in measuring sustainable diets, and there are uncertainties related to “rebound effects” in markets and consumer behaviors (*114*). Culturally, alternative protein sources may be deemed “too radical” for mainstream consumption (*115*).

Seafood, however, is a widely accepted alternative to meat and is rich in protein and micronutrients such as fatty acids. Replacing today’s overfishing with sustainable fishing might meet an additional demand of 20 million metric tons annually (*116*). However, it is recognized that a future sustainable seafood demand must be supplemented by mariculture of organisms at lower trophic levels to maximize efficiency, both as direct food and feed ingredients (*116*–*118*). One of the options to increase food availability (without increasing fishing pressure) is to reorient fisheries from feed production to direct human consumption (*116*). It has been estimated that—depending on policy reforms, technological innovation, and the extent of future shifts in demand—edible food from the sea could be increased by 21 million to 44 million metric tons by 2050, a 36 to 74% increase compared to current yields (*117*). Environment-friendly and sustainable mariculture of unfed organisms has the potential to release pressure on agricultural land, fresh water, fertilizers, and capture fisheries (*116*, *118*, *119*).

#### Barriers and opportunities for implementation in Peru

Considering this intervention in the context of Peru, the average protein intake is 20% lower than the U.S. Department of Agriculture–recommended diet (*120*). Although it may be perplexing to campaign for reduced or limited meat consumption in this context, there could be a strong opportunity for Peru to develop dietary guidelines that address both health and environmental sustainability, thus promoting a more diverse diet with a higher proportion of fruits and vegetables, as well as locally produced food such as quinoa, corn, and potatoes that could contribute to conserving genetic diversity. There remains, however, an important issue that dietary choices are limited in many places, so food options are based on availability rather than preference. Peru’s vast production and export of fishmeal mean that these valuable proteins and essential oils are used as feed worldwide rather than as domestic food. One of the options to increase food availability (without increasing fishing pressure) is to redirect fisheries from feed production (about 20 × 10^6^ metric tons year^−1^) to direct human consumption (*116*). There is considerable potential for some of the Amazon freshwater species to contribute to aquaculture, as is seen in Amazonian Peru (*121*).

#### Barriers and opportunities for implementation in the United Kingdom

Personal and cultural connections with food and issues of food justice and access make actions to influence demand challenging. Tighter restrictions on advertising of unsustainable production of products or overconsumption (particularly of discretionary foods), as well as labeling and awareness campaigns, could support changes in diets (*122*).

#### Implications for targets and indicators

Reductions in animal products should be context specific, and scenarios for shifts in diets should take into account trade-offs between sustainability indicators (*66*). We propose that by 2025, member states develop dietary guidelines that address health and environmental sustainability, promoting a more diverse and nutritionally balanced diet of fruits, vegetables, meat, and seafood. More sustainable diets could be promoted by transforming “reduction” fisheries (i.e., fisheries, often on lower trophic levels, that process their catch into fish meal or fish oil) into fisheries that directly provide food for human consumption by 2025. By 2025, incentives could be developed for increased mariculture of edible sea plants and filter feeders. Addressing child hunger and malnutrition, it is proposed that by 2025, schools provide sustainable and varied meals to children up to the age of 12, following national dietary guidelines. Possible indicators could include the number of countries with dietary guidelines that address both health and sustainability, per capita meat consumption, per capita seafood consumption, and ratio of harvest from reduction fisheries versus total fisheries, as well as national plans for cultivation of maricultured sea plants and filter feeders. There are opportunities to align reporting of progress in meeting these targets and reporting of SDG indicators 12.1.1 (Number of countries with sustainable consumption and production national action plans or sustainable consumption and production mainstreamed as a priority or a target into national policies) and 12.1.2 (Prevalence of moderate or severe food insecurity in the population, based on the Food Insecurity Experience Scale).

### Action 7. Mainstream biodiversity considerations in food systems (cross-cutting)

#### Global level policy findings

We suggest that the preceding six targets, in the context of a number of enabling conditions, will support mainstreaming biodiversity in food systems (Fig. 1). A key barrier to the adoption of biodiversity policies has been their lack of integration in mainstream economic sectors, especially the food system. Consequently, over the past decade, biodiversity mainstreaming has become a key facet of the global conservation and sustainable development agendas. Such mainstreaming requires the inclusion of biodiversity considerations into policies, strategies, and practices of key public and private actors that affect or rely on biodiversity, so that biodiversity is conserved and sustainably used, both locally and globally (*10*). The SDGs, Aichi Targets, and proposed post-2020 targets mention integrating biodiversity values into national and local planning, development processes, and poverty reduction strategies and accounts [SDG 15.9, Aichi Target 2, and CBD updated Zero Draft E 12. (c) 13]. However, most CBD member states have not adopted National Biodiversity Strategies and Action Plans as policies integrating all relevant economic sectors (*123*).

#### Barriers and opportunities for implementation in Peru and the United Kingdom

The Peruvian Government’s strategy to address deforestation and climate change has a high potential for delivering beneficial outcomes for biodiversity (*124*). Despite this potential, specific biodiversity targets are not incorporated into sector strategies, national development planning, impact assessment evaluations, or budgets. As a result, biodiversity policies are disconnected from sectoral policies (*125*). Progress in relation to the actions discussed would have important benefits to mainstreaming efforts. In combination with strengthened governance (discussed next), and enabling conditions by diverse agents of change, these actions could support transformative change in relation to food production and consumption.

### Action 8. Strengthen governance of sustainable food production and consumption (cross-cutting)

#### Global level policy findings

It was apparent from our analysis that strengthening governance for sustainable food production and consumption is needed, both within and beyond the scope of the CBD (Fig. 1). Within the CBD, substantial efforts are needed to agree to SMART international and national targets to support implementation, create a system of robust and transparent reporting, and improve accountability and enforcement mechanisms. Furthermore, CBD governance should ensure inclusive processes involving multiple stakeholders. Beyond the scope of the CBD, the effective implementation of sustainable food production and consumption actions will require wider enabling conditions and the action of diverse agents of change (Table 4, discussed in the following section).

#### Barriers and opportunities for implementation in Peru and the United Kingdom

Although National Biodiversity Strategies and Action Plans constitute the primary means of implementation of the CBD and are intended to trigger the creation of concrete policy instruments, their ambition and alignment with the 2010–2020 strategic plan have been often lacking (*126*, *127*). In Peru, the National Biodiversity Strategies and Action Plans have lacked alignment with the Aichi targets, and implementation has been slow (*128*). In the United Kingdom, each administration has implemented National Biodiversity Strategies and Action Plans, some with national targets, but there has been a lack of genuine political commitment to biodiversity policies at the national level.

#### Implications for strengthening governance within the CBD

Although slow implementation is partly a result of the lack of measurable indicators, National Biodiversity Strategies and Action Plans have been criticized for being “mere declarations of intention” rather than firm commitments to action (*129*), lacking accountability and compliance (*130*, *131*). Furthermore, there are no sanctions if member states fail to fulfill their obligations (*132*), the dispute mechanism has never been used, and there is no compliance committee.

In the absence of official CBD feedback on individual state progress, nongovernmental organizations (NGOs) have taken the lead to review and report on member state progress (*131*). The CBD’s Global Biodiversity Outlooks report on overall status and biodiversity trends based on national reports, National Biodiversity Strategies and Action Plans (NBSAPs), and other sources. The Subsidiary Body on Implementation, which started in 2016, could have a more active role in evaluating implementation. It could also propose innovative means of compliance, for example, through financial or trade sanctions (such as through the Convention on International Trade in Endangered Species under which countries risk trade sanctions if found in serious noncompliance) or “naming and shaming” to increase global ambition (such as in the Paris Agreement of the United Nations Framework Convention on Climate Change where individual countries have made voluntary pledges, allowing for comparison and review of each member state’s performance).

The fact that countries agreed to a compliance committee as part of the Paris Climate Agreement (*133*) shows that this may be within reach of the CBD. Furthermore, countries could agree to less harsh, but more innovative means of promoting compliance. For example, increased transparency in relation to state progress could take the form of a “naming but not shaming” approach that supports member states struggling to reach their goals. For example, the NBSAP voluntary peer review mechanisms could become compulsory (*131*). Currently, the pilot voluntary peer review mechanism is being piloted in Ethiopia and India (*134*, *135*). The voluntary nature contrasts with the compulsory United Nations Framework Convention on Climate Change (UNFCCC) Measurement, Reporting and Verification system and the Universal Periodic Review of the UN Human Rights Council. To make progress on CBD compliance, we suggest that the post-2020 biodiversity framework further develops a voluntary peer review mechanism and adopts a compulsory review mechanism linked to a formal review of individual member state performance. Thereby, countries most needing support to implement CBD obligations can be targeted for peer review and exchange of best practices as well as financial and capacity building support (*131*).

There is evidence that inclusive multistakeholder processes at all levels of CBD governance can help achieve mainstreaming of biodiversity policies (*136*) within government and business. At the national level, it is important to consider the concerns of all relevant actors and to support NBSAP implementation when internalizing international legal obligations into national policy, thus reducing ambiguity and ensuring accountability.

## ENABLING ACTIONS AND SUPPORT BY DIVERSE AGENTS OF CHANGE

The notion of mainstreaming requires careful consideration of the multiple tensions that exist between goals as well as the ways in which economic development projects undermine other sustainability objectives. For example, large-scale development plans will have important implications on biodiversity (e.g., governments are expected to spend US$60 trillion on new infrastructure globally by 2040) (*137*). Furthermore, despite their ambition, we believe that the targets proposed in this paper will be insufficient for achieving the sustainable production and consumption of food necessary to protect biodiversity. The fulfillment of a transformative post-2020 biodiversity agenda transcends the mandate of the CBD framework as it currently exists and requires broader enabling conditions to ensure greater compliance, transparency, and accountability of the activities of incumbent actors and industries (Table 3). The development of enabling conditions requires redressing power through the actions of a broad range of agents (Table 4). The actions of these agents either in collaboration or individually can support the speed of change, such as was observed in the (eventual) action to address ocean plastics (*138*) or when actors are mobilized around a common theme, such as youth-led climate justice movements calling for bold action by governments.

From a practical perspective, greater support will be needed for gathering the necessary indicator data. Here, there are opportunities for synergies. For instance, Peru has set up a forest monitoring system for the UNFCCC, and both agriculture and environment ministries are using one system. In 2019, the Ministry of Environment of Peru developed a map on ecosystem degradation for the United Nations Convention to Combat Desertification, which can also be used to monitor habitat changes.

While multistakeholder collaboration and negotiations are frequently considered normative aspects of sustainability governance, it is important, and in many instances challenging, to ensure that negotiations and participatory practices between actors are meaningful. To that end, all actions need to be linked to broader, long-term processes of empowerment (*139*). This mobilization of diverse agents for change helps to secure long-term outcomes for biodiversity, by directly including those who affect or are affected by (un)sustainable food production and consumption, and can support long-term positive changes that are not reversed when funding runs out, the priorities of private owners change, or land ownership changes (*140*). Our analysis has shown that action for changing values, the adoption of standards with biodiversity considerations, and changes to agricultural and fishing practices are highly influential, and could have a long-lasting impact, in bending the curve of biodiversity loss. Tools, such as Life Cycle Assessment and sustainability standards, if pursued in the context of good governance including accountability and transparency, could support the post-2020 global biodiversity framework.

## CONCLUSIONS

Although $44 trillion (over half) of the world’s gross domestic product (GDP) is highly or moderately dependent on nature (*141*), our review of eight actions demonstrates that it is extremely challenging to “decouple” biodiversity loss and development based on current models that prioritize economic growth at the expense of multiple social-ecological values. The proposed targets will only be effective if states, the private sector, and civil society exhibit the political will to achieve them. The targets, if implemented effectively and in the context of the wider enabling conditions identified, will reduce terrestrial habitat loss (attaining zero net loss) and ensure sustainable fisheries (conservation and sustainable use of species), which together will help attain the CBD’s goal of living in harmony with nature. However, the changes needed to value and mainstream biodiversity will be highly dependent on actions outside the CBD, in combination with action and alignment with other bodies and frameworks. These require synergies with the SDGs, which, in turn, will be necessary to attain the CBD’s 2050 vision.

We have intentionally specified a short time frame for progress against the proposed targets (by 2025), as immediate implementation is required that will demand practical steps to be taken related to the availability of data and resources, and institutional arrangements. Only the highest level of ambition in setting and implementing the goals will support the CBD’s 2050 target of living in harmony with nature (*16*). Fundamentally, there is a need to open up discussions and possibilities for more sustainable and equitable economic models, which might include redefining GDP—or a “green GDP”—to ensure that development is fully connected to well-being and nature. Although these would constitute major measures, transformative change requires bold actions by diverse agents in food systems to meaningfully address current trends in biodiversity loss.

## Supplementary Material

http://advances.sciencemag.org/cgi/content/full/7/12/eabc8259/DC1

Adobe PDF - abc8259_SM.pdf

Actions on sustainable food production and consumption for the post-2020 global biodiversity framework

## SUPPLEMENTARY MATERIALS

Supplementary material for this article is available at http://advances.sciencemag.org/cgi/content/full/7/12/eabc8259/DC1
