# Supplementary material for: Actions on sustainable food production and consumption for the post-2020 global biodiversity framework
Source: Sci Adv. 2021 Mar 19;7(12):eabc8259. doi: 10.1126/sciadv.abc8259 (PMC7978425; doi:10.1126/sciadv.abc8259)
Supplement: http://advances.sciencemag.org/cgi/content/full/7/12/eabc8259/DC1 [file supp_7_12_eabc8259__7.12.eabc8259.DC1.html]

Science Advances | Science AdvancesAAASSearchScience AdvancesMenu

## Supplementary Materials

# Actions on sustainable food production and consumption for the post-2020 global biodiversity framework

Izabela Delabre, Lily O. Rodriguez, Joanna Miller Smallwood, Jörn P. W. Scharlemann, Joseph Alcamo, Alexander S. Antonarakis, Pedram Rowhani, Richard J. Hazell, Dag L. Aksnes, Patricia Balvanera, Carolyn J. Lundquist, Charlotte Gresham, Anthony E. Alexander, Nils C. Stenseth

Download Supplement

**This PDF file includes:**

- Fig. S1
- Tables S1 to S4

**Files in this Data Supplement:**

- Adobe PDF - abc8259\_SM.pdf
