# Supplementary material for: Actions on sustainable food production and consumption for the post-2020 global biodiversity framework
Source: Sci Adv. 2021 Mar 19;7(12):eabc8259. doi: 10.1126/sciadv.abc8259 (PMC7978425; doi:10.1126/sciadv.abc8259)
Supplement: http://advances.sciencemag.org/cgi/content/full/7/12/eabc8259/DC1 [file supp_7_12_eabc8259__abc8259_SM.pdf]

Science Advances
AAAS

advances.sciencemag.org/cgi/content/full/7/12/eabc8259/DC1

Supplementary Materials for

Actions on sustainable food production and consumption for the post-2020 global biodiversity framework

Izabela Delabre*, Lily O. Rodriguez, Joanna Miller Smallwood, Jörn P. W. Scharlemann, Joseph Alcamo, Alexander S. Antonarakis, Pedram Rowhani, Richard J. Hazell, Dag L. Aksnes, Patricia Balvanera, Carolyn J. Lundquist, Charlotte Gresham, Anthony E. Alexander, Nils C. Stenseth*

*Corresponding author. Email: i.delabre@bbk.ac.uk (I.D.); n.c.stenseth@ibv.uio.no (N.C.S.)

Published 19 March 2021, *Sci. Adv.* **7**, eabc8259 (2021)
DOI: 10.1126/sciadv.abc8259

This PDF file includes:

Fig. S1
Tables S1 to S4

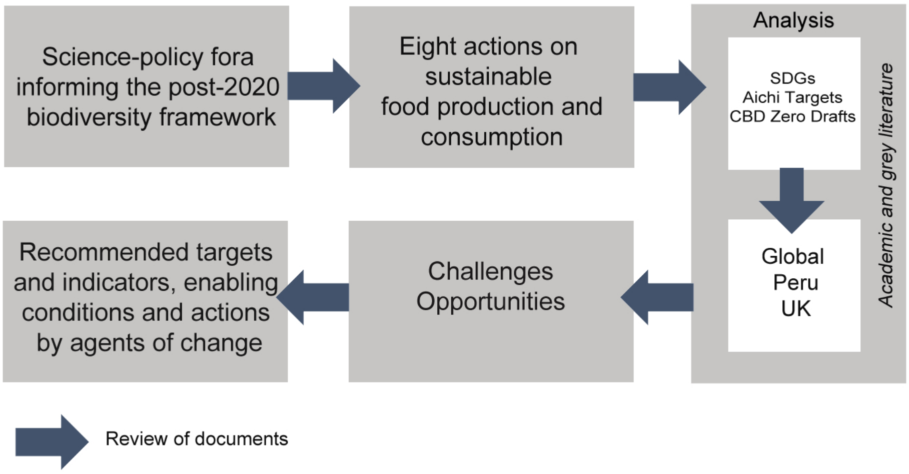

Fig. S1. Research approach for analyzing actions proposed in four science-policy fora and their potential implementation

Table S1. Details and scope of science-policy fora informing the post-2020 biodiversity framework

Science-policy forum	Date and location	Forum details
IPBES visioning workshop: New visions for nature and nature's contributions to people for the 21st century	4-8 September 2017 Auckland, New Zealand	Workshop participants included a total of 73 participants from inter-governmental organizations, national government organizations, academia and the private sector. Participants were from 31 countries, and held a range of sectoral expertise on biodiversity topics, from urban development to agriculture and fisheries. At this week-long workshop participants developed seven positive future visions for nature, including detailed narrative texts that discussed what the future looks like in a particular narrative, and key aspects or actions (Social, Technological, Economic, Environmental, Political and Value) that are required to achieve a particular future vision.
IUBS 4th Science Forum: Towards living in harmony with nature by 2050: A Science-Policy Dialogue	23-24 November 2018 Sharm El-Sheikh, Egypt (CBD COP 14)	Science-policy dialogue organized by the International Union of Biological sciences, the European Commission (with support from EKLIPSE, the EU science-policy interface mechanism on biodiversity and ecosystem services), the Inter-American Institute for Global Change Research, and the Secretariat of the Convention on Biological Diversity. Brought together and engaged scientists and policy makers to discuss how science can inform the post-2020 global biodiversity framework. The discussions particularly focused on what scientists could offer to the preparation of the post-2020 agenda. Reflected on concepts for transition, on the role of scenarios and pathways, as well as on scale and scope of actions towards the 2050 vision.
IUBS Science Forum, dialogue at the IUBS Centenary Year 33rd General Assembly and Scientific Program	31 July 2019 Oslo, Norway	This transdisciplinary science session discussed ideas, themes and indicators to inform the post-2020 global biodiversity framework related to sustainable production and consumption. It discussed the interlinkages between food production and consumption and the SDGs, targets and indicators related to sustainable production and consumption; and potential targets for the post-2020 biodiversity framework, and the actions required by governments, business and stakeholders at all levels to ensure effective, accurate and coherent reporting against indicators.
Ninth Trondheim Conference on Biodiversity	2-5 July 2019 Trondheim, Norway	The conference gathered around 450 participants, with representatives registered from almost 120 countries. Participation in the Conference was by invitation only. In addition to country representatives, there was participation from UN bodies, international and non-governmental organizations, as well as to other major groups and stakeholders. The conference sought to facilitate a shared understanding of key knowledge areas, and to help ensure that the process for developing the post-2020 global biodiversity framework is knowledge-based, just and inclusive.

Table S2. Detailed actions proposed during four science-policy fora informing the post-2020 biodiversity framework

Proposed actions	Proposed actions reflected in 'short list' of key actions							
	1. Remove harmful incentives	2. Accounting for true value	3. Food waste reduction	4. Sustainability standards	5. Life cycle assessments	6. Sustainable diets	7. Mainstream	8. Strengthen governance
Engagement of citizens in biodiversity issues to promote its sustainable use		■	■	■	■	■	■	■
Environmental education at all levels (with teaching on local food diversity, school gardens, with biodiversity and ecosystem services in university textbooks)			■	■		■	■	
Advisory services for small-scale producers			■	■	■		■	■
Agricultural systems shift to local, cultural, away from massive monocultures	■	■		■	■	■	■	■
Agroforestry systems	■	■		■	■	■	■	■
Multifunctional/diverse agricultural landscapes; use of diverse and well-adapted crop varieties; 10% of buildings' energy and food needs supplied through green infrastructure; urban agroecological gardens	■	■		■	■	■	■	■
Promoting sustainable increases in the productivity of existing agricultural land and rangeland	■			■			■	■
Moratoria on soya and meat produced on recently-cleared land	■			■	■	■	■	■
Ecological restoration in agricultural landscapes		■		■	■	■		■
All plant-based material is produced from 100% sustainable sources				■		■	■	
Giving priority to farming native species, so as to avoid possible to invasions of native habitats by escaped alien species, and species lower down the food chain (e.g. herbivorous fish rather than carnivores). This can be achieved through a combination of regulations and promoting changes in consumer preferences		■				■		■
Promoting public policies and incentives that maintain local varieties of crops and indigenous breeds in production systems	■						■	■
Increased adoption of good agricultural practices; Improved targeting and efficiency of fertilizer, pesticide and water use; No till techniques	■			■	■		■	■
Climate smart agriculture	■			■	■		■	■
Sustainable use of varieties of crops and trees, their wild relatives, and breeds of livestock; gene banking					■			
Recognising the multiple values of biodiversity in the strategies and processes that drive decisions about development		■					■	■
Reduce demand for endangered species consumption/ derived from illegal killing and trade					■	■	■	■
Biodiversity in climate change funding		■					■	■
Encouraging a landscape matrix that equally supports nature and biodiversity/ systematic spatial planning changes the value system to a "green-attitude"		■		■			■	
Biodiversity-specific requirements to be taken into account by public agencies when applying for funding		■					■	
Collective land-ocean governance vision (e.g., IPBES and other intergovernmental process lead the way to the establishment of an Oceans Council)							■	■

Strengthening the biodiversity component of emerging voluntary sustainability initiatives such as standard-setting and certification within international supply chains								
Realizing the full potential of emerging sustainability standards and certification								
Further development of certification schemes to fill current gaps								
Organic certification and conservation agriculture								
Community co-management of fisheries								
Indigenous and local communities are actively involved in the management and restoration of the coasts (including, for example, participating in community coral gardening).								
Governance and responsibility for land management to indigenous and local communities (respected, subject to national legislation and relevant international obligations)								
Increasing levels of food sovereignty and food production in the hands of small farmers								
Community gardens								
Decentralized networks (farmer field schools, Trainer-to-Trainer programmes)								
Empower IPLC to implement nature-based solutions								
Improved access to finance (especially for women) for food production and biodiversity conservation and sustainable use								
Conservation and sustainable use in corporate sustainability plans								
Fewer non-renewable resources, sustainable use of renewable resources. Increasing production intensity to lower footprint								
Public (consumer) awareness programs (environmental, ecological and nutrition consciousness)								
Leveraging the power of consumer choice by emphasizing the health and cost benefits of choices that also benefit biodiversity								
Tools to trace individuals' footprints to influence consumption decisions								
Prices that reflect the scarcity of natural resources as well as the environmental impact of farming can contribute to greater efficiency, polluter pays principle enforced through charges and regulations								
Eating artificially produced fish protein, food produced from waste products or eating across the food chain								
Dietary guidelines - credible information on the environmental impact of their food choices								
Addressing shifts in consumption patterns, including moderate meat consumption, veganism, synthetic meat.								
Slow food movement, eating according to the season, locally sourced food								
Gathering more data and establishing harmonized indicators to measure effectiveness and track progress of policies on sustainable consumption and production								
Scientific data and results are publicly available, in a form useable by policy makers, other researchers and society								
Developing the voluntary peer review system already being used at the CBD (under monitoring and enforcement)								
Participatory monitoring, control, enforcement and surveillance systems for a sustainable management of ecosystems and their benefits (e.g., forest and fisheries)								
By 2030 we reduce by 25% the ecological footprint of food production and consumption and reduce by 50% by 2050. Concept of 'food print'								
Circular economy concept								

Pricing the externalities of food production/consumption waste to drive production/consumption systems that maximize quality and accessibility, minimize impact on biodiversity and minimize cost								
Recharacterisation of gross domestic product (GDP) “growth” to ensure it is connected to well-being and nature. Including metrics such as biodiversity, quality of life and natural resource use (metric contrasting natural capital and consumption) e.g., Bhutan happiness index, taxation.								
Green GDP can be a key tool for measuring progress and guiding decisions around sustainable use.								
Internalizing environmental costs and getting the price right is necessary to reach and maintain sustainable food production systems and consumption. Economists valuing internalised ecological and social features.								
Improved valuation, accounting and reporting of biodiversity and ecosystem services (national accounting systems capturing economic, cultural, social, intergenerational growth).								
International natural resource consumption taxation system that redistributes funds to a common international funding pool to alleviate poverty, support environmental management, and provide venture capital for sustainable technological innovation.								
Green/ resource use taxation								
High seas are closed to fishing								
Enhancing, in each country, monitoring and enforcement of regulations to prevent illegal, unregulated and unreported fishing by flag-vessels								
Fishing at or within maximum sustainable yield (MSY)								
Phasing out fishing practices and gear which cause serious adverse impacts to the seafloor or to non-target species								
Governance that crosses the land-sea interface (e.g., Arctic Council, cumulative effects)								
Coastal zones are managed sustainably (ban of unsustainable fishing practices).								
Guidelines of fisheries and other sectors to become OECMs								
Removal, phase-out, or reform of harmful incentives (fishing encouraging overcapacity, removing or reforming bio-energy subsidies)								
Use of positive incentives e.g. subsidies rewarding farming practices that safeguard the environment, for switching to organic agriculture or integrated practices (public money for public goods), subsidies help people in impoverished areas to be able to offer their locally sourced products								
Negative incentives for “non-compliance” with sustainable production requirements								
Incentives to align sector activities with biodiversity conservation and sustainable use								
Pay attention to the relationship between tenurial rights to agriculture and conservation/develop relevant framework to address this lack of effective tenure systems								
Legal or policy framework for land use or spatial planning. Integrated land-use planning, strategic environmental assessment								
Use information tools as a basis for setting land use policy that takes account of the needs of multiple agendas while maintaining the essential ecological functions								
Action to require the industry to exclude deforesters from their supply chains: addressing commodity supply chains to restrict products from illegal or unsustainable sources								
Regulations (including production protocols) in driving more informed choices								

Zoning system includes a strict no-take zone coinciding with ancient sacred areas								
Reducing waste and losses in supply chains								
Short circuit food provision: e.g., 'la rouche qui dit oui' in France which cuts out the middleman in food production and supply								
Blockchain and similar technologies to improve traceability of commodities and highlight unsustainable trade								
Twitter, cloud-based applications, QR-codes etc. could provide a more detailed picture for consumers								
Use of ‘multitrophic aquaculture’ in which seaweed can be produced for human food, fish feed and pharmaceuticals, reducing feed demand and pollution (e.g, eating invasive species, algae, jellyfish).								
Sustainable and cheaply produced artificially grown fish protein, as well as manufactured food from waste products (e.g., fish skeletons)								
Using waste from one species to be converted to protein by another species, thereby reducing nutrient pollution								
High-tech and traditional agrotechnologies are applied to the agro-food system to maximize ecosystem services								
Technological innovations, co-developed with producers, with researchers and industry; Aquaponics production systems								
Innovative trade relations: Countries will need to negotiate the potential decreases in trade in certain exotic types of food.								
Create a system of transparent life-cycle assessments supporting wise and sustainable consumption and production. Governments and businesses account for the full life cycle of their products								
Track and tracing systems of environmental and social features of food (self-evaluation of food production management and practices, rather than third party accounting); particularly for value chains involving disjunctions between consumption, production and waste disposal								
Increased transparency of public budget								
Financial funding flows have to be made transparent. There must be a mechanism to have users contribute to the implementation of the target. Have the users of Natural Resources (Agriculture, Industry) assist in developing the target, so that they have a part in achieving the target. Some sectors are actually showing that they would like to surpass existing targets								
Set goal in relation to monitoring, control, enforcement and surveillance systems								
Investing in marketing and storage infrastructure and low tech solutions to eliminate food waste								
Minimizing pollution by improving management practices, for example by reducing overfeeding								
Waste streams are managed (bycatch, food waste, life-cycle analysis, et.)								

Table S3. Actions related to sustainable food production and consumption in the SDGs, Aichi Targets and CBD Zero Drafts

Actions	SDGs	Aichi Targets	CBD Zero Draft	CBD Updated Zero Draft
1. Remove incentives that make food production and consumption harmful to biodiversity	2.b Correct and prevent trade restrictions and distortions in world agricultural markets, including through the parallel elimination of all forms of agricultural export subsidies and all export measures with equivalent effect, in accordance with the mandate of the Doha Development Round. 14.6 By 2020, prohibit certain forms of fisheries subsidies which contribute to overcapacity and overfishing, eliminate subsidies that contribute to illegal, unreported and unregulated fishing and refrain from introducing new such subsidies, recognizing that appropriate and effective special and differential treatment for developing and least developed countries should be an integral part of the World Trade Organization fisheries subsidies negotiation.	3. Incentives, including subsidies, harmful to biodiversity, eliminated, phased out or reformed in order to minimize or avoid negative impacts. Positive incentives for conservation and sustainable use of biodiversity developed and applied.	D. 12. (c) 12. Reform incentives, eliminating the subsidies that are most harmful for biodiversity, ensuring by 2030 that incentives, including public and private economic and regulatory incentives, are either positive or neutral for biodiversity.	E. 12. (c) 17. By 2030, redirect, repurpose, reform or eliminate incentives harmful for biodiversity, including [X] reduction in the most harmful subsidies, ensuring that incentives, including public and private economic and regulatory incentives, are either positive or neutral for biodiversity.
2. Accounting for true value and true costs of production by sector	15.9 By 2020, integrate ecosystem and biodiversity values into national and local planning, development processes, poverty reduction strategies and accounts.	2. Biodiversity values integrated into national and local planning processes, incorporated into national accounting, as appropriate, and into reporting systems	D. 12. (c) 13. Integrate biodiversity values into national and local planning, development processes, poverty reduction strategies and accounts, ensuring by 2030 that biodiversity values are mainstreamed across all sectors and that biodiversity-inclusive strategic environmental assessments and environmental impact assessments are comprehensively applied.	E. 12. (c) 13. By 2030, integrate biodiversity values into policies, regulations, planning, development processes, poverty reduction strategies and accounts at all levels, ensuring that biodiversity values are mainstreamed across all sectors and integrated into assessments of environmental impacts.
3. Reduce food waste and loss	12.3 By 2030, halve per capita global food waste at the retail and	Not explicitly mentioned. Other relevant target: 4.	Not explicitly mentioned. Other relevant targets:	Not explicitly mentioned. Other relevant targets:

across supply chains	consumer levels and reduce food losses along production and supply chains, including post-harvest losses.	Governments, business and stakeholders at all levels have taken steps to achieve or have implemented plans for sustainable production and consumption, and have kept the impacts of use of natural resources well within safe ecological limits.	D. 12. (b) 8. Conserve and enhance the sustainable use of biodiversity in agricultural and other managed ecosystems to support the productivity, sustainability and resilience of such systems, reducing by 2030 related productivity gaps by at least [50%].<br>D. 12. (c) 14. Reform economic sectors towards sustainable practices, including along their national and transnational supply chains, achieving by 2030 a reduction of at least [50%] in negative impacts on biodiversity.<br>D. 12. (c) 17. People everywhere take measurable steps towards sustainable consumption and lifestyles, taking into account individual and national cultural and socioeconomic conditions, achieving by 2030 just and sustainable consumption levels.	E. 12. (b) 9. By 2030, support the productivity, sustainability and resilience of biodiversity in agricultural and other managed ecosystems through conservation and sustainable use of such ecosystems, reducing productivity gaps by at least [50%].<br>E. 12. (c) 14. By 2030, achieve reduction of at least [50%] in negative impacts on biodiversity by ensuring production practices and supply chains are sustainable.<br>E. 12. (c) 15. By 2030, eliminate unsustainable consumption patterns, ensuring people everywhere understand and appreciate the value of biodiversity, and thus make responsible choices commensurate with 2050 biodiversity vision, taking into account individual and national cultural and socioeconomic conditions.
4. Strengthen sustainability standards and certification	Not explicitly mentioned. Other relevant targets:<br>2.4 By 2030, ensure sustainable food production systems and implement resilient agricultural practices that increase productivity and production, that help maintain ecosystems, that strengthen capacity for adaptation to climate change, extreme weather, drought, flooding and other disasters and that progressively improve land and soil quality.<br>12.6 Encourage companies, especially	Not explicitly mentioned. Other relevant targets:<br>4. Governments, business and stakeholders at all levels have taken steps to achieve or have implemented plans for sustainable production and consumption, and have kept the impacts of use of natural resources well within safe ecological limits.<br>6. By 2020 all fish and invertebrate stocks and aquatic plants are managed and harvested sustainably, legally and applying ecosystem based	Not explicitly mentioned. Other relevant targets:<br>D. 12. (b) 8. Conserve and enhance the sustainable use of biodiversity in agricultural and other managed ecosystems to support the productivity, sustainability and resilience of such systems, reducing by 2030 related productivity gaps by at least [50%].<br>D. 12. (c) 14. Reform economic sectors towards sustainable practices, including along their national and transnational supply chains, achieving by 2030 a reduction of at least [50%] in negative impacts on biodiversity.<br>D. 12. (c) 17. People everywhere take measurable steps towards sustainable	Not explicitly mentioned. Other relevant targets:<br>E. 12. (b) 9. By 2030, support the productivity, sustainability and resilience of biodiversity in agricultural and other managed ecosystems through conservation and sustainable use of such ecosystems, reducing productivity gaps by at least [50%].<br>E. 12. (c) 14. By 2030, achieve reduction of at least [50%] in negative impacts on biodiversity by ensuring production

	large and transnational companies, to adopt sustainable practices and to integrate sustainability information into their reporting cycle.<br>14.4 By 2020, effectively regulate harvesting and end overfishing, illegal, unreported and unregulated fishing and destructive fishing practices and implement science-based management plans, in order to restore fish stocks in the shortest time feasible, at least to levels that can produce maximum sustainable yield as determined by their biological characteristics.	approaches, so that overfishing is avoided, recovery plans and measures are in place for all depleted species, fisheries have no significant adverse impacts on threatened species and vulnerable ecosystems and the impacts of fisheries on stocks, species and ecosystems are within safe ecological limits.<br>7. By 2020 areas under agriculture, aquaculture and forestry are managed sustainably, ensuring conservation of biodiversity.	consumption and lifestyles, taking into account individual and national cultural and socioeconomic conditions, achieving by 2030 just and sustainable consumption levels.	practices and supply chains are sustainable.<br>E. 12. (c) 15. By 2030, eliminate unsustainable consumption patterns, ensuring people everywhere understand and appreciate the value of biodiversity, and thus make responsible choices commensurate with 2050 biodiversity vision, taking into account individual and national cultural and socioeconomic conditions.
5. Promote the use of life cycle assessments	Not explicitly mentioned. Other relevant targets:<br>2.4 By 2030, ensure sustainable food production systems and implement resilient agricultural practices that increase productivity and production, that help maintain ecosystems, that strengthen capacity for adaptation to climate change, extreme weather, drought, flooding and other disasters and that progressively improve land and soil quality.<br>8.4 Improve progressively, through 2030, global resource efficiency in consumption and production and endeavour to decouple economic growth from environmental degradation, in accordance with the 10-Year Framework of Programmes on Sustainable Consumption and Production, with	Not explicitly mentioned. Other relevant target: 4. Governments, business and stakeholders at all levels have taken steps to achieve or have implemented plans for sustainable production and consumption, and have kept the impacts of use of natural resources well within safe ecological limits.	Not explicitly mentioned. Other relevant targets:<br>D. 12. (c) 14. Reform economic sectors towards sustainable practices, including along their national and transnational supply chains, achieving by 2030 a reduction of at least [50%] in negative impacts on biodiversity.<br>D. 12. (c) 17. People everywhere take measurable steps towards sustainable consumption and lifestyles, taking into account individual and national cultural and socioeconomic conditions, achieving by 2030 just and sustainable consumption levels.	Not explicitly mentioned. Other relevant targets:<br>E. 12. (b) 9. By 2030, support the productivity, sustainability and resilience of biodiversity in agricultural and other managed ecosystems through conservation and sustainable use of such ecosystems, reducing productivity gaps by at least [50%].<br>E. 12. (c) 14. By 2030, achieve reduction of at least [50%] in negative impacts on biodiversity by ensuring production practices and supply chains are sustainable.<br>E. 12. (c) 15. By 2030, eliminate unsustainable consumption patterns, ensuring people everywhere understand and appreciate the value of biodiversity, and thus make responsible choices commensurate

	developed countries taking the lead.			with 2050 biodiversity vision, taking into account individual and national cultural and socioeconomic conditions.
6. Promote sustainable and varied diets	Not explicitly mentioned. Other relevant targets:<br>2.4 By 2030, ensure sustainable food production systems and implement resilient agricultural practices that increase productivity and production, that help maintain ecosystems, that strengthen capacity for adaptation to climate change, extreme weather, drought, flooding and other disasters and that progressively improve land and soil quality.<br>14.4 By 2020, effectively regulate harvesting and end overfishing, illegal, unreported and unregulated fishing and destructive fishing practices and implement science-based management plans, in order to restore fish stocks in the shortest time feasible, at least to levels that can produce maximum sustainable yield as determined by their biological characteristics.	Not explicitly mentioned. Other relevant targets:<br>6. By 2020 all fish and invertebrate stocks and aquatic plants are managed and harvested sustainably, legally and applying ecosystem based approaches, so that overfishing is avoided, recovery plans and measures are in place for all depleted species, fisheries have no significant adverse impacts on threatened species and vulnerable ecosystems and the impacts of fisheries on stocks, species and ecosystems are within safe ecological limits.<br>7. By 2020 areas under agriculture, aquaculture and forestry are managed sustainably, ensuring conservation of biodiversity.	Not explicitly mentioned. Relevant action target:<br>D. 12. (c) 17. People everywhere take measurable steps towards sustainable consumption and lifestyles, taking into account individual and national cultural and socioeconomic conditions, achieving by 2030 just and sustainable consumption levels.	Not explicitly mentioned. Relevant targets:<br>E. 12. (b) 8. By 2030, ensure benefits, including nutrition, food security, livelihoods, health and well-being, for people, especially for the most vulnerable through sustainable management of wild species of fauna and flora.<br>E. 12. (b) 9. By 2030, support the productivity, sustainability and resilience of biodiversity in agricultural and other managed ecosystems through conservation and sustainable use of such ecosystems, reducing productivity gaps by at least [50%].<br>E. 12. (c). 14. By 2030, achieve reduction of at least [50%] in negative impacts on biodiversity by ensuring production practices and supply chains are sustainable.<br>E. 12. (c) 15. By 2030, eliminate unsustainable consumption patterns, ensuring people everywhere understand and appreciate the value of biodiversity, and thus make responsible choices commensurate with 2050 biodiversity vision, taking into account individual and national cultural and socioeconomic conditions.

7. Mainstream biodiversity considerations in food systems (cross-cutting)	8.4 Improve progressively, through 2030, global resource efficiency in consumption and production and endeavour to decouple economic growth from environmental degradation, in accordance with the 10-Year Framework of Programmes on Sustainable Consumption and Production, with developed countries taking the lead.	4. Governments, business and stakeholders at all levels have taken steps to achieve or have implemented plans for sustainable production and consumption, and have kept the impacts of use of natural resources well within safe ecological limits.<br>19. By 2020, knowledge, the science base and technologies relating to biodiversity, its values, functioning, status and trends, and the consequences of its loss, are improved, widely shared and transferred, and applied.	I. 8. (i) Mainstreaming biodiversity across all sectors of society with a focus on engaging those sectors that will be responsible for implementing actions to address the drivers of biodiversity loss.<br>D. 12. (c) 12. Reform incentives, eliminating the subsidies that are most harmful for biodiversity, ensuring by 2030 that incentives, including public and private economic and regulatory incentives, are either positive or neutral for biodiversity.<br>D. 12. (c) 13. Integrate biodiversity values into national and local planning, development processes, poverty reduction strategies and accounts, ensuring by 2030 that biodiversity values are mainstreamed across all sectors and that biodiversity-inclusive strategic environmental assessments and environmental impact assessments are comprehensively applied.<br>D. 12. (c) 14. Reform economic sectors towards sustainable practices, including along their national and transnational supply chains, achieving by 2030 a reduction of at least [50%] in negative impacts on biodiversity.<br>D. 12. (c) 15. Resources, including capacity-building, for implementing the framework have increased from all sources so that by 2030 resources have increased by [X%] and are commensurate with the ambition of the targets of the framework.<br>D. 12. (c) 16. Establish and implement measures in all countries by 2030 to prevent potential adverse impacts of biotechnology on biodiversity.<br>D. 12. (c) 17. People everywhere take measurable steps towards sustainable consumption and lifestyles, taking into account individual and national cultural and socioeconomic conditions, achieving by	E. 12. (c) 13. By 2030, integrate biodiversity values into policies, regulations, planning, development processes, poverty reduction strategies and accounts at all levels, ensuring that biodiversity values are mainstreamed across all sectors and integrated into assessments of environmental impacts.<br>E. 12. (c). 14. By 2030, achieve reduction of at least [50%] in negative impacts on biodiversity by ensuring production practices and supply chains are sustainable.<br>E. 12. (c). 15. By 2030, eliminate unsustainable consumption patterns, ensuring people everywhere understand and appreciate the value of biodiversity, and thus make responsible choices commensurate with 2050 biodiversity vision, taking into account individual and national cultural and socioeconomic conditions.<br>E. 12. (c) 16. By 2030, establish and implement measures to prevent, manage or control potential adverse impacts of biotechnology on biodiversity and human health reducing these impacts by [X].<br>E. 12. (c) 17. By 2030, redirect, repurpose, reform or eliminate incentives harmful for biodiversity, including [X] reduction in the most harmful subsidies, ensuring that incentives, including

			2030 just and sustainable consumption levels.<br>D. 12. (c) 18. Promote education and the generation, sharing and use of knowledge relating to biodiversity, in the case of the traditional knowledge, innovations and practices of indigenous peoples and local communities with their free, prior and informed consent, ensuring by 2030 that all decision makers have access to reliable and up-to-date information for the effective management of biodiversity.<br>D. 12. (c) 19. Promote the full and effective participation of indigenous peoples and local communities, and of women and girls as well as youth, in decision-making related to the conservation and sustainable use of biodiversity, ensuring by 2030 equitable participation and rights over relevant resources.<br>D. 12. (c) 20. Foster diverse visions of good quality of life and unleash values of responsibility, to effect by 2030 new social norms for sustainability.	public and private economic and regulatory incentives, are either positive or neutral for biodiversity.<br>E. 12. (c) 18. By 2030, increase by [X%] financial resources from all international and domestic sources, through new, additional and effective financial resources commensurate with the ambition of the goals and targets of the framework and implement the strategy for capacity-building and technology transfer and scientific cooperation to meet the needs for implementing the post-2020 global biodiversity framework.<br>E. 12. (c) 19. By 2030, ensure that quality information, including traditional knowledge, is available to decision makers and public for the effective management of biodiversity through promoting awareness, education and research.<br>E. 12. (c) 20. By 2030, ensure equitable participation in decision-making related to biodiversity and ensure rights over relevant resources of indigenous peoples and local communities, women and girls as well as youth, in accordance with national circumstances.
8. Strengthen governance of sustainable food production and consumption (cross-cutting)	2.4 By 2030, ensure sustainable food production systems and implement resilient agricultural practices that increase productivity	4. By 2020, at the latest, Governments, business and stakeholders at all levels have taken steps to achieve or have implemented	8. (d) Reducing time lags in planning, accounting for them in implementation and ensuring effective reviews of progress.<br>8. (f) Ensuring implementation is	G. 14. (a) The participation of indigenous peoples and local communities and a recognition of their rights in the

	and production, that help maintain ecosystems, that strengthen capacity for adaptation to climate change, extreme weather, drought, flooding and other disasters and that progressively improve land and soil quality.<br>8.4 Improve progressively, through 2030, global resource efficiency in consumption and production and endeavour to decouple economic growth from environmental degradation, in accordance with the 10-year framework of programmes on sustainable consumption and production, with developed countries taking the lead.<br>9.4 By 2030, upgrade infrastructure and retrofit industries to make them sustainable, with increased resource-use efficiency and greater adoption of clean and environmentally sound technologies and industrial processes, with all countries taking action in accordance with their respective capabilities.<br>12.2 By 2030, achieve the sustainable management and efficient use of natural resources.<br>16.6 Develop effective, accountable and transparent institutions at all levels.<br>16.7 Ensure responsive, inclusive, participatory and representative decision-making at all levels.<br>16.8 Broaden and strengthen the	plans for sustainable production and consumption and have kept the impacts of use of natural resources well within safe ecological limits.<br>7. By 2020 areas under agriculture, aquaculture and forestry are managed sustainably, ensuring conservation of biodiversity.<br>13. By 2020, the genetic diversity of cultivated plants and farmed and domesticated animals and of wild relatives, including other socio-economically as well as culturally valuable species, is maintained, and strategies have been developed and implemented for minimizing genetic erosion and safeguarding their genetic diversity.<br>17. By 2015 each Party has developed, adopted as a policy instrument, and has commenced implementing an effective, participatory and updated national biodiversity strategy and action plan.<br>18. By 2020, the traditional knowledge, innovations and practices of indigenous and local communities relevant for the conservation and sustainable use of biodiversity, and their customary use of biological resources, are respected, subject to national legislation and relevant international obligations, and fully integrated and reflected in the implementation of the Convention with the full and effective participation of indigenous and local	participatory, inclusive, gender-responsive, transformative, comprehensive, catalytic, visible, knowledge-based, transparent, efficient, results-oriented, iterative and flexible.<br>8. (g) Regular monitoring, evaluation and feedback of the progress towards the attainment of all elements of the framework, including the actions taken, their effectiveness, and resulting changes in biological, social and economic conditions.<br>F. 14. (g) Adequate inclusive and integrative governance is put in place to ensure policy coherence and effectiveness for the implementation the framework.<br>G. 16. (a) Reflecting the framework in relevant planning processes, including national biodiversity strategies and action plans.<br>G. 16. (b) Periodic reporting, including through the use of identified indicators, by Governments, multilateral environmental agreements and other relevant international processes, indigenous peoples and local communities, civil society and the private sector of the actions taken to implement the framework, the successes achieved, and the challenges encountered.<br>Annex I<br>I. B. 3 The framework will be implemented primarily through activities at the national level, with supporting action at the subnational, regional and global levels. It aims to promote synergies and coordination with relevant processes. It provides a global, outcome-oriented framework for the development of national, and as appropriate, regional, goals and targets and, as necessary, the updating of national biodiversity strategies and action plans to achieve these, and to facilitate regular monitoring and review of progress at the global level.	implementation of the framework<br>G. 14. (b) The participation of all relevant stakeholders, non-governmental organizations, youth, civil society, local and subnational authorities, the private sector, academia and scientific institutions through a whole-of-society approach and through inclusive and representative multi-stakeholder and multisectoral platforms;<br>G. 14. (g) Inclusive and integrative governance and whole-of-government approaches to ensure policy coherence and effectiveness for the implementation the framework<br>H. 15. The successful implementation of the framework is dependent on the use of a comprehensive system for planning, reporting and review. It allows for transparent communication of progress to all, rapid course correction and timely input in the preparation of the next global biodiversity framework.<br>H. 16. This system minimizes the burden on Parties, the Secretariat and other entities. It is aligned with, and where appropriate integrated with other processes and other relevant multilateral conventions including Agenda 2030 and the Sustainable Development Goals.<br>H. 17. The system will be complemented by the systems of non-State

	participation of developing countries in the institutions of global governance.	communities, at all relevant levels.		actors, and the development of new systems or the adaptation of existing ones is encouraged. H. 18. The system for planning, reporting and review for national government entities includes the following elements: (a) Planning: (i) National strategies and action plans: a. Are the main instrument to identify national commitments; b. Include all targets and actions; c. Address all performance indicators relevant to the identified targets drawing on the monitoring framework attached to this framework; d. Should include a financing plan; e. Should be updated quickly according to an agreed schedule. (ii) Indicators will be an important part of planning and reporting process including Headline indicators; (iii) Planning documents will be updated on a continuous basis. (b) Reporting: (i) National reports: a. National reports will report on all actions identified in the national strategies and action plans using agreed indicators including headline indicators; b. National reports will be issued regularly and in time, in accordance with the agreed schedule. (ii) Global reports: a. Global stocktake: i. Global collation of statistical information; ii. Takes place frequently according to an agreed schedules. (iii) A reporting schedule that will be used

				consistently by all institutions involved; (iv) Global assessment processes, including the Global Biodiversity Outlook and the Intergovernmental Science-Policy Platform on Biodiversity and Ecosystem Services will be reviewed for efficiency and complementarity and timeliness. (c) Review: (i) Global analysis of progress on objective/numerical elements of targets and progress towards milestones and goals; (ii) [Open ended Forums] for the review of national implementation and to share lessons learned and best practices; (iii) Voluntary in-depth peer review of national implementation by experts including from other parties.

Table S4. Barriers and opportunities relating to implementation of actions proposed in four science-policy fora

**Actions from science-policy fora**	**Level of analysis**	**Barriers**	**Opportunities**
1. Remove incentives that make food production and consumption harmful to biodiversity	Global	• Difficult to identify (lack of transparency).<br>• Difficult to attribute biodiversity loss to particular incentive.<br>• Politically difficult to reform because of strong opposition from recipients.<br>• Tightly linked with regional/international trade.<br>• Impacts on low income and poorly resourced producers.	• Transparency and accountability in subsidy reporting, e.g. effective WTO notification requirements are needed (*58*).<br>• Better monitoring of the impacts of subsidies needed to determine relative benefits.<br>• Value scientific advice of multiple effects of subsidies and subsidy reform.<br>• NGOs/civil society to take up agenda politically.
	Peru	• Tightly linked with regional/international trade, e.g. US-Peru Trade Promotion Agreement.<br>• 2001 Agrarian Promotion Law has allowed the payment of less income tax and a more flexible labor regime for the agricultural sector (which has been reflected in the growth of agribusiness).	• Recognizing the importance of diversified farming systems and diverse suite of options for development pathways, moving away from credit policies that incentivize high-input monoculture and land use conversion (*59*).<br>• Transparency and accountability for land use decisions, including for the private sector (*60*).<br>• Recognise both 'official' and unofficial (or direct/indirect) subsidies.
	UK	• EU Common Agricultural Policy has substantially impacted natural habitats, whilst driving over-production of various commodities (*62*).<br>• Potentially weaker regulations in relation to UK pesticide use (*64*, *65*).	• Post-Common Agricultural Policy UK policy to remove incentives harmful to biodiversity.<br>• Post-Brexit trade discussions to incorporate strong biodiversity/sustainability criteria.
2. Accounting for true value and true costs of production by sector	Global	• Successful implementation is still in early stages. Governmental policies and market transactions typically do not reflect the full value of nature's contributions to people.<br>• Lack of interdisciplinary and transdisciplinary competences to support integration of indigenous and local knowledge into scientific analysis and policy making (*68*).<br>• Non-monetary values that are not amenable to economic methods including other worldviews and associated values are not considered, including those associated with individual and shared socio-cultural values, those underpinned by indigenous local knowledge, as well as other biophysical and health-related values (*68*).	• Strong government support and progressive targets for valuing and investing in natural capital, including non-monetary value/<br>• Strong business commitments, and compliance mechanisms for private actors to support societal long-term goals.<br>• More research into valuation and its effects (precautionary approach).<br>• Connecting science and policy actors, indigenous and local knowledge, appreciating and exchanging respective multidisciplinary knowledges.<br>• Financial and technical resources through effective communication, training and the creation of a network of information and fundraising volunteers.
	Peru	• Natural capital accounting implementation still in its infancy.	• Natural capital accounting could be integrated and measured at national level.

		• Currently not integrated into national information systems and repeatedly measured (*70*).	
	UK	• Measurement of natural capital is difficult due to the lack of a baseline (*72*).<br>• The telecoupled interactions in food systems globally are not considered and biodiversity impacts are displaced in supply chains.	• Natural capital accounting needs to consider the telecoupled interactions in food systems globally as biodiversity impacts are displaced in supply chains.
3. Reduce food waste and loss across supply chains	Global	• Complexity in attribution of food/biofuel production to biodiversity loss (including direct and indirect drivers).<br>• Rapid urbanization and globalization, means that food supply chain require adequate roads, transportation and marketing infrastructure.<br>• Shifts towards items with short shelf life associated with food waste (*75*).	• Changes in consumers' perceptions of food waste<br>• Advocacy and campaigns<br>• Closed loop supply chain models<br>• Application of food waste hierarchy<br>• Advertising rules to limit overconsumption<br>• Ban landfill of organic waste<br>• Advisory bodies on waste minimization and research to support<br>• Multiple actor dialogue to identifying and mobilizing key stakeholders to address food waste throughout supply chains, including producer support.<br>• Redistribution of food, food sovereignty and access
	Peru	• Demands of supermarkets for certain food product criteria.<br>• Unequal food distribution and access.	• Law No. 30988 passed to address food loss and waste across Peru's food supply chain.<br>• Improving nature of processing, logistics and warehousing from farm to consumer.
	UK	• Demands of supermarkets for certain food product criteria.<br>• Relatively low food prices.<br>• Retailer power encourages overspending.<br>• Food waste management through anaerobic digestion may disincentivize food waste reduction.	• SDG target of halving per capita global food waste at retail and consumer levels by 2030.
4. Strengthen sustainability standards and certification	Global	• Impacts of certification on biodiversity contested; some evidence of positive impacts (*83*, *84*).<br>• MSC certified areas mainly in Global North.<br>• Small proportion of total area of farmland covered by certification schemes; lowest cost compliance.<br>• Focus on small number of commodities.<br>• Reliance on developed country consumption; certified production outweighs market demand.<br>• Driven by private actors - disconnect with policy objectives.	• Strong regulation and enforcement of local laws and complementarity<br>• Government agencies act as facilitators and moderators of multi-stakeholder processes, involving MNCs, local institutions and farmers<br>• Stronger accountability mechanisms/auditing processes<br>• Use of mitigation hierarchy<br>• Recognition by standard schemes of landscape diversity<br>• Stronger systems for monitoring, reporting, enforcement, credibility, traceability and accountability<br>• NGOs/CSOs to push for stronger standards.

		• Need to find appropriate costing model for internalizing biodiversity protection.<br>• Inconsistent criteria in standards; lack of comparability.	• Consumers to signal demand for more sustainable products.<br>• Incentives for targeted certification where biodiversity is most threatened.
	Peru	• High costs of certification (documentation, labor costs)(*94*).<br>• Small-scale farmer exclusions.<br>• Elite capture.<br>• High labor and input costs, volatile cocoa prices and limited options to diversity on-farm income.<br>• Lack of enforcement of legal prescription about land use change and allocation of land rights.	• Stronger systems for monitoring, reporting, enforcement, credibility, traceability and accountability.<br>• Focus on inclusiveness through more participatory approaches in environmental impact assessment/HCV/HCSA assessments within certification requirements.<br>• Strong regulation and enforcement of local laws and complementarity.<br>• Opportunity of landscape/ emerging 'jurisdictional approaches' to support more joined up governance.
	UK	• National supermarkets have an important role in food spending and sustainability certification.	• Use of standards by regulators, public procurement policies.<br>• Import and export taxes for sustainable trade.<br>• Commensurate effort by demand-side supply chain actors.
5. Promote the use of life cycle assessments	Global	• Lack of clarity of what goes into biodiversity footprint (*98*, *100*).<br>• Current Life Cycle Assessment methodologies lack the spatial resolution and predictive ecological information to reveal key impacts on climate, water and biodiversity<br>• Bias: Life Cycle Assessment may favor high-input intensive agricultural systems and misrepresent less intensive or smaller-scale agroecological systems (*97*).<br>• Complexity in capturing all impacts e.g. for adjacent/indirectly connected areas.<br>• Challenge for Life Cycle Assessments to tackle transparency and accountability of agricultural products and fisheries for opaque supply chains.<br>• Exclusions of some supply chain actors due to costs imposed and principles of equity and justice largely absent.<br>• Data collection burden: who monitors, evaluates/audits.	• Biodiversity reporting and methodology needs to be standardized and transparent.<br>• Regulation for labelling on products to make Life Cycle Assessment data transparent to all stakeholders down to the consumer.<br>• Dialogues across multiple stakeholders, NGO, Government, farmers, business and transport in creating standardized labelling for key Life Cycle Assessment impacts, reporting, and accountability frameworks.<br>• Focus on inclusiveness and cost-effective mechanisms for reporting, especially for small-scale farmers.<br>• Use of Life Cycle Assessment in green public procurement.<br>• Biodiversity values recognized to support shift towards demand for more sustainable products.
	Peru	• Lack of research into Life Cycle Analysis and biodiversity impacts of commodities and products.	• Peru has recently opened a Centre for Life Cycle Analysis as part of a Life Cycle Initiative, providing momentum and attention to this area of research.
	UK	• Uncertainty in current methodologies pertaining to	• UK has set up a working group, a methodology aiming to drive

		telecoupled impacts e.g. the source of feed in cattle production.	biodiversity benefits and climate mitigation.<br>• Consultation on regulation for due diligence on deforestation-risk commodities (October 2020).
6. Promote sustainable and varied diets	Global	• Meat and dairy industry lobbying.<br>• Subsidies supporting unsustainable production and consumption.<br>• Uneven patterns in consumption; access and food justice.<br>• Restricted choice in many contexts (subsistence agriculture and 'food deserts').<br>• Alternative protein sources deemed 'too radical' for mainstream (*115*).<br>• Lack of uptake of issue by environmental groups (*112*, *113*).<br>• Complex measurement of sustainable diet.<br>• Possible rebound effect of switching to vegetarian diet (*114*).	• Alignment with health guidelines.<br>• Transparency and accountability on meat and associated feed production.<br>• Increased awareness of biodiversity issues associated with meat production.<br>• NGO/environmental group campaigning, e.g. Friends of the Earth Europe Meat Atlas.<br>• Political will to drive change.<br>• Redirection of harmful subsidy income to low-income healthy diet shifts.
	Peru	• Dietary choices are limited in many places, food options are based on availability rather than preference.<br>• Uneven patterns in consumption; access and food justice.	• Labels providing information regarding environmental and health issues (*120*).<br>• Dietary guidelines that address both health and environmental sustainability, recognizing cultural embedded nature of eating practice and current inequalities.<br>• Redirect fisheries from feed production to direct human consumption.
	UK	• Meat and dairy industry lobbying<br>• Subsidies supporting. unsustainable production and consumption.<br>• Uneven patterns in consumption; access and food justice.	• Dietary guidelines that address both health and environmental sustainability, recognizing cultural embedded nature of eating practice and current inequalities.<br>• Tighter restrictions on advertising of unsustainable production of products or excessive consumption.
7. Mainstream biodiversity considerations in food systems (cross-cutting)	Global	• Biodiversity policies are not integrated in mainstream economic sectors in the food system (agriculture, fisheries and aquaculture).<br>• Lack of appreciation of diverse visions of good quality of life, focus on economic growth as measured by GDP.<br>• Lack of effective communication and siloed approaches.<br>• Lack of policy integration.<br>• Colonial policies reflected in regulations and corporate practices focused on extraction.<br>• Supply chain complexity and opacity.	• Political will to change.<br>• Transparency and accountability in transactions and supply chains.<br>• Appreciation of local and traditional ecological knowledge in science-policy discussions.<br>• Open up policy discussions on diverse sustainable development pathways.<br>• Appreciation of environmental justice concerns.

	Peru	• Specific biodiversity targets are not incorporated into sector strategies, national development planning, impact assessment evaluations, or budgets, resulting in biodiversity policies being disconnected from sectoral policies.	• Restoration of terrestrial and/or coastal ecosystems with climate, biodiversity, equity and justice concerns as a requirement in development plans.<br>• Diversification of agroecosystems.
	UK	• Specific biodiversity targets are not incorporated into sector strategies, national development planning, impact assessment evaluations, or budgets, resulting in biodiversity policies being disconnected from sectoral policies.	• Restoration of terrestrial and/or coastal ecosystems with climate, biodiversity, equity and justice concerns as a requirement in development plans.<br>• Diversification of agroecosystems.
8. Strengthen governance of sustainable food production and consumption (cross-cutting)	Global	• Lack of ambition.<br>• Lack of accountability and compliance (*130*, *131*).<br>• Trade-offs between competing interests.<br>• Limited connectivity and collaboration between stakeholders in decision-making processes, and the alignment of vision and objectives across institutions.<br>• Lack of implementation.	• Increased ambition of shared understandings at the CBD COP (state and non-state actors) (*131*, *136*).<br>• Increased transparency in relation to individual country progress towards the CBD's new targets, and of the CBD reporting mechanism (*131*).<br>• CBD Compliance committee to sanction states, conduct peer review and strengthen compliance (*130*).<br>• Make use of the CBD voluntary dispute resolution mechanism.<br>• Use of NBSAPS to implement Aichi Targets, and to link to other biodiversity conventions and related SDGs.
	Peru	• Lack of connectivity between different related regimes in natural resource management (*125*).<br>• National Biodiversity Strategies and Action Plans have lacked alignment with the Aichi targets, and implementation has been slow (*128*).	• Compliance, accountability and transparency in post-2020 biodiversity framework.<br>• High ambition on sustainable food production and consumption
	UK	• National Biodiversity Strategies and Action Plans lacking accountability and compliance.	• Compliance, accountability and transparency in post-2020 biodiversity framework.<br>• High ambition on sustainable food production and consumption.
